# Acetone-mediated ammonium oxidation to dinitrogen by *Zobellella taiwanensis* bacteria

**DOI:** 10.1093/ismejo/wraf230

**Published:** 2025-10-27

**Authors:** Yu Lei, Yangqing Wang, Xiaojuan Tan, Chuanwu Xi, Hong Liu

**Affiliations:** Chongqing Institute of Green and Intelligent Technology, Chinese Academy of Sciences, Chongqing 400714, China; College of Resources and Environment, Chongqing School, University of Chinese Academy of Sciences, Chongqing 400714, China; Chongqing University, Chongqing 400044, China; Chongqing Institute of Green and Intelligent Technology, Chinese Academy of Sciences, Chongqing 400714, China; College of Resources and Environment, Chongqing School, University of Chinese Academy of Sciences, Chongqing 400714, China; Chongqing University, Chongqing 400044, China; Anhui Provincial Key Laboratory of Molecular Enzymology and Mechanism of Major Metabolic Diseases, College of Life Sciences, Anhui Normal University, Wuhu 241000, China; Department of Environmental Health Sciences, University of Michigan, Ann Arbor, MI 48109, United States; Chongqing Institute of Green and Intelligent Technology, Chinese Academy of Sciences, Chongqing 400714, China; College of Resources and Environment, Chongqing School, University of Chinese Academy of Sciences, Chongqing 400714, China

**Keywords:** ammonium oxidation, N_2_ production, AMAO process, Zobellella taiwanensis

## Abstract

Bioconversion of ammonium to dinitrogen (N_2_) is an essential process in the nitrogen cycle, primarily driven by O_2_-dependent nitrification and followed by O_2_-limited denitrification, involving multiple redox states of nitrogen (NH_4_^+^ → NH_2_OH → NO_2_^−^ → NO_3_^−^ → NO_2_^−^ → NO→N_2_O → N_2_). Here, we discovered a new process termed acetone-mediated ammonium oxidation in *Zobellella taiwanensis* bacteria under both oxic and anoxic conditions, directly oxidizing ammonium to N_2_ (NH_4_^+^ + acetone → acetoxime → N_2_ + acetone). Acetone, produced from organic sources, couples with ammonium to form acetoxime in the presence of O_2_, NO_2_^−^, NO_3_^−^, or Fe(III). Subsequently, acetoxime is oxidized to N_2_, thereby releasing recyclable acetone. We purified two new enzymes (acetoxime synthase; acetoxime dehydrogenase) catalyzing this process and identified their corresponding genes. The widespread distribution of homologous amino acid sequences across thousands of prokaryotic and eukaryotic microorganisms suggests the potential ubiquity of this process in nature and its possible substantial contributions to the nitrogen cycle.

## Graphical Abstract

**Graphical Abstract:**
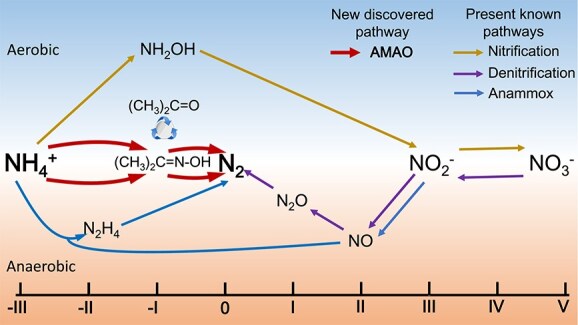

## Introduction

Bioconversion of ammonia to N_2_ is an essential process for returning nitrogen from the geobiosphere to the atmosphere [1]. This process underpins our understanding of nitrogen dynamics on both global and local scales, with applications in agricultural nitrogen management and wastewater treatment [2, 3]. To date, two well-established pathways for this process have been identified. One is the earliest and primary pathway, involving a combination of nitrification (NH_4_^+^ → NO_3_^−^), driven by autotrophic nitrifiers, and denitrification (NO_3_^−^ → N_2_), carried out by heterotrophic denitrifiers. The other is anaerobic ammonium oxidation (anammox), where autotrophs anaerobically oxidize ammonium to N_2_ using nitrite as an electron acceptor [4].

Currently, an increasing number of heterotrophic bacteria have been reported to autonomously convert ammonium to N_2_, suggesting a potential alternate pathway by heterotrophs [5]. However, the mechanisms and environmental significance of this process remain poorly understood [5]. Simultaneous heterotrophic nitrification and aerobic denitrification is commonly suggested, supported by the production of nitrite or nitrate during this process and the presence of denitrifying genes [5, 6]. However, many of these bacteria produce little or no nitrite and nitrate [7], even after addition of denitrification inhibitors [8]. Furthermore, the multi-step oxidation of NH_4_^+^ (–III N) to NO_3_^−^ (+V N), followed by its reduction to N_2_ (0 N), requires a total of 13 electron transfers per nitrogen atom. Such an extensive redox cascade may impose significant metabolic costs, as each step demands dedicated enzymes and potential energy inputs (denitrification typically depends on electron donors generated from organic carbon, while the energy conservation mechanisms in heterotrophic nitrification remain unclear) [9].

Recent studies on *Alcaligenes* species have introduced new insights. A pathway termed “direct ammonium oxidation” (dirammox) has been proposed, in which ammonium is converted to hydroxylamine that is then directly oxidized to N_2_ [8]. However, uncertainties remain regarding the production and fate of hydroxylamine [9], with a study suggesting that its conversion to N_2_ may be an abiotic process [10]. Moreover, the key gene cluster *dnfABC* [8], implicated in dirammox, is absent in many other heterotrophic bacteria capable of converting ammonium to N_2_ (*Z. taiwanensis*, *Aeromonas* sp*.*, *Klebsiella pneumoniae*, *Vibrio diabolicus*, *Photobacterium* sp*.*, *Halomonas* sp*.*, *Paracoccus pantotrophus*, *Agrobacterium* sp., *Cupriavidus* sp*.*, *Diaphorobacter* sp*.*, *Arthrobacter* sp*.*, *Microbacterium* sp*.*, *Bacillus subtilis*, and *Exiguobacterium* sp*.*) [11–24], indicating that alternative pathways may exist.

Despite advances, a lack of concrete evidence persists regarding the specific proteins and genes related to ammonium oxidation by heterotrophs [25]. Furthermore, it is believed that heterotrophs cannot obtain energy to support microbial growth via these pathways but instead consume assimilable organic matter and ammonium [26]. Additionally, heterotrophic ammonium oxidation is typically considered an aerobic process, yet observations in natural ecosystems suggest that ammonium oxidation and N_2_ production under anoxic conditions may also be linked to heterotrophic activity [27]. These unresolved questions highlight the need to elucidate the mechanisms of the heterotrophic ammonium-to-N_2_ conversion.

*Zobellella taiwanensis* DN-7, a heterotrophic bacterium belonging to *Gammaproteobacteria*, has demonstrated an ability to convert ammonium into N_2_ [11]. During this process, only trace amounts of nitrite (NO_2_^−^-N < 0.5% of initial ammonium-N) were detected, with no measurable accumulation of hydroxylamine, nitrate, nitric oxide, or nitrous oxide. This unique product profile suggests the potential existence of a pathway distinct from conventional processes.

In this study, we investigate *Z. taiwanensis* to provide new insights into the mechanisms of heterotrophic ammonium-to-N_2_ conversion through integrated physiological, isotopic, enzymatic, and genomic analyses. We elucidate the key intermediates, mass balance and molecular mechanisms underlying this pathway, and demonstrate this conversion process under anoxic conditions. Furthermore, we preliminarily evaluate its environmental implications through phylogenetic distribution analysis of the key enzymes and genes.

## Materials and methods

### Strains, media, and isotope-labeled materials

Basic media (BM) were prepared following the reference with slight modifications [11]. The modified BM composition per liter included: 0.66 g (NH_4_)_2_SO_4_, 5.72 g C_6_H_5_Na_3_O_7_•2H_2_O (sodium citrate), 0.4 g K_2_HPO_4_, 0.02 g MgSO_4_•7H_2_O, and 0.1 ml trace element solution, with the pH adjusted to 8.5–9.0 using NaOH solution. *Zobellella taiwanensis* DN-7 was grown under oxic conditions on a rotary shaker at 30°C in the modified BM. Under anoxic conditions, sucrose was used as the carbon source instead of citrate, and nitrate, nitrite, and Fe(III) were added as electron acceptors based on experimental requirements. *Escherichia coli* EPI300-T1 was used for fosmid construction, and *E. coli* DH5α was used for heterologous expression. All solutions were added to the autoclaved medium through sterile filtration using 0.22 μm pore-size cellulose acetate filters. All experiments were conducted at least in triplicate. Labeled compounds including (^15^NH_4_)_2_SO_4_, Na^15^NO_2_, Na^15^NO_3,_ and ^15^NH_2_OH·HCl (purity >98%) were purchased from the Shanghai Research Institute of Chemical Industry Co., Ltd. The ^15^NO (purity >98%) stock solution was prepared by adding H_2_SO_4_ to a solution containing ^15^N-labeled NO_2_^−^ and KI, capturing the evolved NO gas in anoxic water [28]. The ^15^N_2_O (purity >98%) gas was prepared in the headspace of a helium-flushed sealed bottle containing copperized cadmium and Na^15^NO_3_ at pH 4.7 [29]. ^15^N-labeled acetoxime (purity >98%) was synthesized using acetone and ^15^NH_2_OH·HCl as reactants, as described in the reference [30].

### Physiological experiments with *Z. taiwanensis*

#### Inhibitor experiments

For inhibitor experiments, *Z. taiwanensis* was treated with 0.1 mM sodium diethyldithiocarbamate (DDC) or 5% (v/v) acetylene (Supplementary methods).

#### Acetone and acetoxime production dynamics

To detect acetone and acetoxime production during ammonium oxidation underoxic conditions, *Z. taiwanensis* was inoculated into 500 ml triangular flasks containing 200 ml of BM (10 mM (NH_4_)_2_SO_4_) and incubated at 30°C and 180 rpm. For anoxic experiments, *Z. taiwanensis* was inoculated into 340 ml sealed bottles containing 50 ml of BM, which was flushed with pure argon for at least 15 min before inoculation. The BM contained 5 mM (NH_4_)_2_SO_4_ and 10 mM NaNO_2_ for nitrite experiments, as well as 10 mM NaNO_3_ or 50 mM ferric citrate for relevant experiments. The bottles were incubated at 30°C for 72 h (NaNO_2_), 96 h (NaNO_3_), or 144 h (ferric citrate) with agitation in the dark. Samples (1 ml) for chemical analyses were taken during the incubations for both oxic and anoxic experiments. To evaluate the effect of ferric forms on ammonium oxidation, Fe_2_(SO_4_)_3_, Fe(OH)_3_, Fe_2_O_3,_ and ferric citrate were added to the BM at final concentrations of 25, 50, 25, and 50 mM, respectively (undissolved compounds were considered dissolved). Cultures of 5% *Z. taiwanensis* were inoculated and incubated under anoxic conditions as previously described. After incubation, ammonium was measured, and the percentage of ammonium consumption for the initial ammonium was calculated. Ammonium concentration effects were examined with 5 mM pyruvate-C and varying ammonium-N levels (0.5, 5, 50 mM) under oxic and anoxic conditions, maintaining a nitrate-N/ammonium-N ratio of 0.6 (with nitrate as electron acceptor) for anoxic cultures.

#### Substrates of acetoxime synthesis

To investigate the substrates involved in acetoxime synthesis, different combinations of nitrogen and carbon sources (hydroxylamine alone, hydroxylamine with pyruvate, ammonium with acetone, ammonium with pyruvate, and hydroxylamine with acetone) were examined. *Zobellella taiwanensis* cells were harvested and resuspended in 10 ml of BM solution at an optical density of OD_600_ = 3. The BM contained 2 mM of nitrogen compounds (ammonium or hydroxylamine) and carbon compounds (acetone or pyruvate) while depleting ammonium sulfate and sodium citrate. The cell suspension was then transferred to a 100 ml triangular flask and incubated at 30°C and 150 rpm for 2 h. Liquid samples were collected, centrifuged, and the concentration of acetoxime in the supernatant was monitored. This process was repeated to investigate the involvement of 10 mM ammonium and 15 mM acetone as reactants in acetoxime formation, with measurements taken at various time points up to 16 h. pH values were recorded at the beginning and end of each experiment.

#### Products of acetoxime oxidation

To determine the products of acetoxime oxidation by *Z. taiwanensis*, 10 mM acetoxime was added to a sealed 100 ml bottle of BM supplemented with a gas mixture of He/O_2_ = 1:1. After incubation, air samples from the headspace were collected to measure levels of CO_2_, N_2_, NO, and N_2_O. Liquid samples were analyzed for acetone, pyruvate, NO_2_^−^, and NO_3_^−^. A replication experiment was conducted to investigate acetone generation during acetoxime oxidation, with ammonium sulfate and sodium citrate depleted in the BM. pH values were monitored throughout the experiment.

#### Source of acetone production

To identify the source of acetone production by *Z. taiwanensis*, cells were resuspended in 10 ml of BM solution depleted of citrate to an OD_600_ of 3. Various carbon sources containing 10 mM C, including glucose, sucrose, citrate, fumarate, succinate, acetate, pyruvate, lactate, and acetoxime, were added separately to the BM solution. Acetone concentrations were measured after a 6-h incubation at 30°C and 150 rpm. To explore the direct precursor for acetone formation, 2 mM of sodium acetoacetate, 2-propanol, and hydroxyacetone were inoculated in 50 ml of BM solution containing 1 mM of pyruvate and 10 mM of ammonium as sole carbon and nitrogen sources. The group without these added compounds was used as control. After a 1% inoculation of *Z. taiwanensis* cultures, the bottles were incubated at 30°C and 150 rpm. Acetone concentrations were measured at 4, 8, 15, 23, and 26 h. Additionally, the utilization of acetone was tested by inoculating 1% *Z. taiwanensis* cultures in BM containing acetone as the sole carbon source instead of citrate. Similar experiments were carried out using acetoxime as the sole nitrogen or carbon source in the BM to assess acetoxime utilization by *Z. taiwanensis*.

#### ^15^N_2_ production analysis

For ^15^N_2_ production analysis, ^15^N-labeled NH_4_^+^ or acetoxime was treated with O_2_, NO_2_^−^, NO_3_^−^, or Fe(III) (ferric citrate) using *Z. taiwanensis* cells. Unlabeled NH_4_^+^ or acetoxime was treated with ^15^N-labeled NO_2_^−^ or NO_3_^−^. The cells were harvested and resuspended in 10 ml of BM solution, depleted of ammonium sulfate and sodium citrate, to an OD_600_ of 3. The cell suspension was then transferred to sealed 140 ml bottles. Various samples, with different combinations (Supplementary Table S1), were prepared and added to the testing BM solution. All nongaseous components listed above, except for Fe(III) (5 mM), have a concentration of 2.5 mM. Negative controls were performed without *Z. taiwanensis* to exclude the influence of abiotic reactions. The net ^15^N_2_ production in experimental vials was calculated by subtracting the ^15^N_2_ value measured in the no-bacteria controls from the ^15^N_2_ value of each sample vial. Anoxic conditions were achieved using pure He, while oxic conditions were achieved using a 1:1 mixture of He/O_2_. The prepared bottles were then incubated at 30°C for 4 h. One milliliter of gas sample from the headspace was taken and injected into a 12 ml exetainer (Labco) that was filled with helium for ^15^N_2_ measurement.

### Purification of acetoxime synthase and acetoxime dehydrogenase

To prepare the cell-free extract, *Z. taiwanensis* was incubated in modified BM containing 70 mM ammonium with the addition of 20 mM acetone. Cells were harvested at an OD_600_ of 5–6 and centrifuged at 8000 *g* for 20 min at 4°C. The pelleted cells were then resuspended in a 50 mM Tris–HCl (pH 8.5) buffer and disrupted by ultrasonication at 200 W for ~15 min in an ice-water bath. After centrifugation at 30 000 *g* for 30 min at 4°C, the supernatant was collected as the AOH crude enzyme fraction. It was rapidly frozen in liquid nitrogen and stored at −80°C. The remaining cell debris was resuspended and washed in 50 mM Tris–HCl (pH 8.5) buffer thrice, followed by centrifugation at 12000 g and 4°C to remove soluble proteins. The precipitate was then resuspended in a 50 mM Tris–HCl (pH 8.5) buffer containing 1.0% dodecyl-β-D-maltoside and incubated at 4°C in the dark for 1 h. After centrifugation at 30 000 *g* and 4°C for 30 min, the supernatant was used as the AOS crude enzyme fraction. It was likewise flash frozen in liquid nitrogen and stored at −80°C.

All subsequent procedures were carried out at a temperature range of 4°C–10°C. The crude enzyme fraction of AOS was applied onto a DEAE-Sepharose Fast Flow column (1.6 cm × 10 cm, Coolaber, China) that had been pre-equilibrated with 50 mM Tris–HCl buffer (pH 9.0). After washing with 50 mM Tris–HCl buffer (pH 9.0) containing 0.5 M NaCl and re-equilibrating with 50 mM Tris–HCl buffer (pH 9.0), the enzyme was eluted using a linear gradient from 0 to 0.5 M NaCl, again in the same buffer containing 0.1% dodecyl-β-D-maltoside. The active fractions were collected and concentrated through ultrafiltration (Millipore Co, USA) and further purified using a gel-filtration column (Superdex Hiload 16/60200 pg, GE Healthcare, USA). This column was eluted with 50 mM Tris–HCl + 150 mM NaCl +0.1% dodecyl-β-D-maltoside at pH 9.0. Each fraction was collected, and the AOS activity was assayed. The concentrated fractions obtained through ultrafiltration represented the purified AOS, and additional purification steps were deemed unnecessary.

For the purification of AOH, the buffer was initially replaced with 50 mM phosphate buffer at pH 7.0 using ultrafiltration. Then, the crude enzyme fraction of AOH was filtered through a 0.4 μm membrane and passed through a Q-Sepharose Fast Flow column (1.6 cm × 10 cm, Coolaber) that had been pre-equilibrated with 50 mM phosphate buffer (pH 6.2). After washing with the same buffer, the eluent containing the unadsorbed fractions was applied to a CM sepharose CL-6B column (1.6 cm × 10 cm, Coolaber) pre-equilibrated with 50 mM phosphate buffer (pH 7.4). Elution was achieved using a linear gradient from 0 to 0.5 M NaCl. Active fractions were pooled and further purified using a gel-filtration column (Superdex Hiload 16/60200 pg). The mobile phase consisted of 50 mM phosphate buffer and 150 mM NaCl at pH 7.4, and active fractions were collected. The concentrated active fractions represented the purified AOH. The protein concentration was determined using a BCA Protein Assay Kit (Beyotime, China).

### Enzyme activity assays

Activity of AOS and AOH was determined by measuring the increase in absorption at 340 nm. One unit is defined as the amount of enzyme that catalyzes the reduction of 1 μmol NAD^+^ per minute. For the AOS assay, the reaction mixture (200 μl) consisted of 50 mM Tris–HCl (pH 8.5), 5 mM NH_4_Cl, 5 mM acetone, 2 mM NAD^+^, and the enzyme preparation. To initiate the reaction, acetone was added to the mixture. The reaction mixture was then incubated at 30°C. Similarly, for the AOH assay, the reaction mixture (200 μl) included 50 mM Tris–HCl (pH 8.5), 10 mM acetoxime, 2 mM NAD^+^, and the enzyme preparation. The reaction was started by adding acetoxime to the mixture. The mixtures were incubated at 30°C. In general, the culture time for both AOS and AOH reactions ranges from 15 min to 1.5 h according to experiment design, and the reactions were stopped by incubating them at 80°C for 5 min, except for the experiments that require detection of acetone. The effects of alternative electron acceptors including FMN, FAD, 2,6-dichloroindophenol sodium salt hydrate (DCIP), NADP^+^, and cytochrome c (cytC, horse heart) were examined at equivalent concentrations to NAD^+^. After reaction, the increase and decrease of acetoxime amounts were measured in AOS and AOH tests, respectively.

To detect acetoxime synthesis by AOS, 3 ml reaction mixtures containing 2 mM ^15^NH_4_Cl, 3 mM acetone, 6 mM NAD^+^, and 5.6 μg purified AOS were incubated at 30°C. One hundred microliters of liquid samples were collected into 1.5 ml tubes and incubated at 80°C for 5 min to stop the enzyme reaction. Next, 1 ml of dichloromethane was added and homogeneously mixed to extract acetoxime, and 800 μl of the dichloromethane samples were filtered using 0.22-μm filters. The filtered samples were stored in vacuum tubes (Agilent) for C_3_H_7_^15^NO detection. To compensate for liquid losses due to sampling, 100 μl of buffer was refilled each time, and the dilution effect of the additional liquid brought into the tube was considered during data processing. To detect acetoxime oxidation by AOH, enzymatic assays were carried out in 2 ml reaction mixtures with 2 mM C_3_H_7_^15^NO, 5 mM NAD^+^, and ~3.7 μg purified AOH. The mixture was directly injected into a 12 ml exetainer that was filled with helium and incubated at 30°C without agitation in the dark. ^15^N_2_ release in the headspace was measured. Other substrates or products were also determined for mass balance calculation in both tests.

### Characterization of acetoxime synthase and acetoxime dehydrogenase sequences

Genomic DNA of *Z. taiwanensis* was sequenced by PacBio SMRT technology (Supplementary methods). A genomic fosmid library of *Z. taiwanensis* was prepared in pCC1FOS following the manufacturer’s protocol (Epicentre, USA). Fosmid clones were selected and preserved in 96-well plates containing 150 μl Luria–Bertani (LB) supplemented with 12.5 μg ml^−1^ chloramphenicol and stored at −80°C in 20% glycerol. Upon activation, the clones were transferred to new 96-well plates with 150 μl modified BM (comprising 1 mM glucose, 20 mM acetone, and 50 mM ammonium) for the initial screening. Clones exhibiting a faster growth rate in the modified BM were further screened by transferring them to two separate LB media, both supplemented with 12.5 μg ml^−1^ chloramphenicol. One LB medium contained 10 mM acetone and 10 mM ammonium, while the other contained 10 mM acetoxime. Following overnight oxic culture at 30°C, the cells were harvested for testing AOS and AOH activity as described earlier. The remaining supernatant was utilized to measure acetoxime levels and pH. Positive clones demonstrating AOS and AOH activity were screened and sequenced using specific pCC1FOS sequencing primers (Epicentre) at BGI (Wuhan, China). The genomic locations of the inserted DNA fragments were determined by blasting against the genome of *Z. taiwanensis*.

Screened potential segments of *aohA*, *aosA*, and *hypA* were amplified from genomic DNA of *Z. taiwanensis* using the primer pairs listed in Supplementary Table S2. Plasmid DNA was prepared using the TIANprep Mini Plasmid Kit (Tiangen, China). PCR amplification was performed using the PrimeSTAR MAX DNA Polymerase (Takara, Japan). Purification of DNA fragments from the PCR reaction was carried out using the Gel & PCR Clean Up Kit (Omega, USA). To construct plasmids pBAD- *aohA*, pBAD- *aosA*, and pBAD- *hypA*, the pBAD/HisA vector (Ampr; Invitrogen, USA) was linearized using primers pBAD-F/pBAD-R. Segments of *aohA*, *aosA*, and *hypA* were amplified from genomic DNA of *Z. taiwanensis* using the primer pairs listed in Supplementary Table S2. The amplified DNA fragments and the linearized vectors were ligated and circularized using the Multi One Step Cloning Kit (Qizyme, China) to generate the clone plasmids.

Subclones containing *aohA*, *aosA*, or *hypA* were characterized for AOS or AOH activity. *Escherichia coli* DH5α containing the vector pBAD/HisA was used as a negative control under the same conditions. For characterization experiments, 100 μl overnight culture clones were inoculated into 100 ml flasks containing 10 ml LB with 10 mM acetoxime or 10 mM acetone and 5 mM ammonium, respectively. Zero-point one percent L-arabinose was injected as an inducer. After incubating at 37°C and 200 rpm for 16 h, the samples were centrifuged. The precipitate was washed and used to measure AOS and AOH activity, and the supernatant was used to detect acetoxime. Beyond heterologous expression, the sequences of purified AOS and AOH were also characterized by liquid chromatography–mass spectrometry (LCMS) (Supplementary methods). The mass spectrometry data were searched against a database of predicted sequences from positive clones obtained earlier. For phylogenetic analyses of AOS and AOH, homologous sequences were retrieved from the National Center for Biotechnology Information (NCBI) database. Neighbor-joining trees were constructed using MEGA 11.

### Analytical methods

The OD_600_ value was measured using a spectrophotometer. NH_4_^+^, NH_2_OH, N_2_, N_2_O, NO, NO_2_^−^, and NO_3_^−^ were determined as described previously [11]. Fe(II) and Fe(III) were monitored using the ferrozine method [31]. Qualitative and quantitative analyses of acetone and acetoxime were performed by gas chromatography (GC; Agilent 7890A, USA) using a DB-WAX column (30 m × 0.32 mm × 0.25 μm) and a flame ionization detector [32]. Citrate and sucrose were measured by high-performance liquid chromatography [33]. Pyruvate was determined using the 2, 4-dinitrophenylhydrazine method [34]. CO_2_ amounts were measured using GC with a flame ionization detector [35]. The amount of ^15^N_2_ was measured using a GasBench II-253 plus isotope ratio mass spectrometer (Thermo Fisher Scientific) with an automated gas preparation unit (GasBench II, Thermo Fisher Scientific). The ^15^N abundance of N_2_O was analyzed by gas chromatograph–isotope ratio mass spectrometry (Thermo Fisher Scientific Delta V Plus, USA) [29, 36]. C_3_H_7_^15^NO was extracted from the mixture using dichloromethane and analyzed using a gas chromatograph–mass spectrometer (7890A-5977A, Agilent, USA) equipped with an HP-5MS fused-silica capillary column (30 m × 0.25 mm × 25 μm, Agilent) [37]. Additional details regarding ^15^N measurements are provided in the Supplementary methods.

## Results

### Discovery of the key intermediate (acetoxime) for the ammonium-to-N_2_ conversion under both oxic and anoxic conditions

To identify the exact pathway of the ammonium-to-N_2_ conversion by *Z. taiwanensis*, we evaluated whether known ammonium transformation pathways (nitrification–denitrification and dirammox) could account for the observed N_2_ production. The genome sequencing of Z. *taniwanensis* revealed that ammonium monooxygenase (AMO) [38, 39], hydroxylamine dehydrogenase (HAO) [38], pyruvic oxime dioxygenase (POD, responsible for pyruvic oxime oxidation to nitrite and pyruvate) [40], and dinitrogen-forming (DNF, responsible for hydroxylamine oxidation to N_2_) [41] like genes were absent, but the complete denitrification genes (*napA, nirK, norB, and nosZ*) were present. Inhibitor experiments (Supplementary Fig. S1) with DDC (copper chelator, AMO and NirK inhibitor) [42] and acetylene (AMO and NosZ inhibitor) [43, 44] both revealed that the ammonium oxidation was not inhibited, and <1% ammonium was converted via nitrification–denitrification, indicating that inorganic nitrogen compounds (NH_2_OH, NO_2_^−^, NO_3_^−^, NO, and N_2_O) involved in nitrification–denitrification pathways were unlikely the primary intermediates in ammonium-to-N_2_ conversion (Supplementary information).

**Figure 1:**
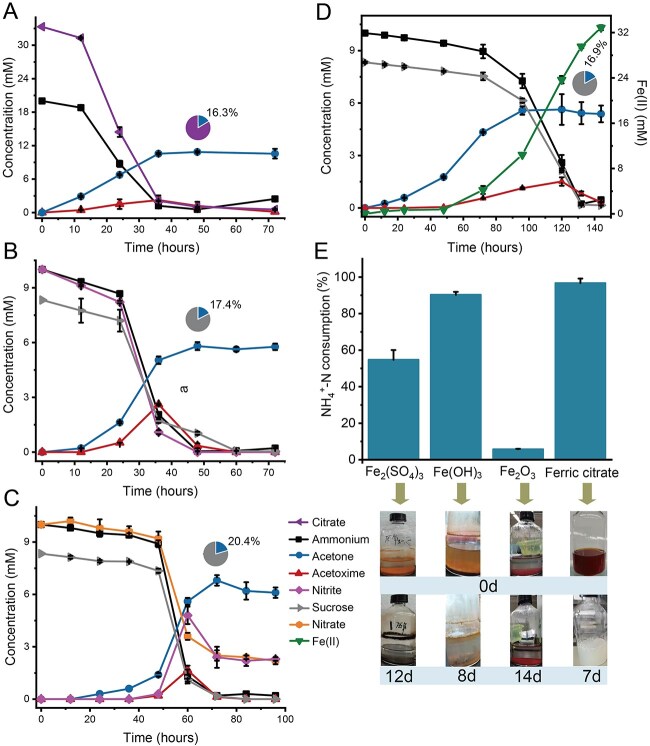
AMAO process by *Z. taiwanensis* under both oxic and anoxic conditions. The percentage of acetone production relative to citrate (A) or sucrose (B–D) consumption is shown in pie charts. (A) Consumption of 20 mM ammonium in the presence of 33.3 mM citrate with the accumulation of acetone and transient acetoxime under oxic conditions. (B–D) Consumption of 10 mM ammonium with the accumulation of acetone and transient acetoxime in the presence of 8.3 mM sucrose and different electron acceptors, including 10 mM nitrite (B), 10 mM nitrate (C), and 50 mM ferric citrate (D) under anoxic conditions. (E) Effects of Fe_2_(SO_4_)_3_, Fe(OH)_3_, Fe_2_O_3_, and ferric citrate on ammonium consumption by *Z. taiwanensis*. Culture time is shown in the grayish-blue bar graphs. Data points show means, while error bars show ±1 s.d. of *n* = 3 biological replicates.

To investigate the possibility of an organic nitrogen intermediate, we specifically examined the organic compounds in several −20°C stored *Z. taiwanensis* cultures from the above acetylene inhibition experiments and observed an unexpected, significant accumulation of acetone (Supplementary Fig. S2B). Subsequent time-course experiments (33.3 mM citrate, 20 mM NH₄^+^) under oxic conditions showed a transient accumulation of acetoxime (up to 13.1% of the added ammonium) in parallel to the production of acetone (Fig. 1A), suggesting that acetoxime might be the key intermediate. In this acetoxime-detected experiment, both citrate and ammonium were consumed concurrently, with a final growth yield (OD_600_) of 1.43 ± 0.15. The buildup of acetone appeared to stem from both the rapid oxidation of acetoxime and continuous acetone production from citrate, which accounted for up to 16.3% of consumed citrate. This substantial acetone output from the citrate-rich medium (C/N = 10) likely supports the growing cellular demand for ammonium oxidation.

It was already known that *Z. taiwanensis* can use NO_2_^−^, NO_3_^−^, or Fe(III) as electron acceptors for growth [11]. Therefore, we proposed that ammonium-to-N_2_ conversion might be coupled to NO_2_^−^, NO_3_^−^, or Fe(III) reduction under anoxic conditions. Indeed, when fed with sucrose, ammonium, and any of these electron acceptors, both transient acetoxime production and acetone production, up to 16.9%–20.4% of the consumed sucrose, were observed as before (Fig. 1B–D). Concurrently, production of nitrite and Fe(II) demonstrated nitrate and Fe(III) reduction. *Zobellella taiwanensis* was also able to reduce ferric sulfate, ferric citrate, and ferric hydroxide (Fig. 1E), as well as insoluble ferric hydroxide (Fig. 1E).

To assess the influence of ammonium concentration on this acetoxime-associated ammonium oxidation, we cultured *Z. taiwanensis* with 5 mM pyruvate-C and varying ammonium-N levels (0.5, 5, and 50 mM) under both aoxic and anoxic conditions (Supplementary Fig. S3). In all setups, pyruvate and ammonium were co-consumed, with growth yields (OD_600_) of 0.38 ± 0.02, 0.25 ± 0.01, and 0.26 ± 0.01 for oxic cultures (0.5, 5, and 50 mM NH_4_^+^-N, respectively) and 0.03 ± 0.00, 0.12 ± 0.02, and 0.32 ± 0.05 for anoxic cultures. These results suggested that ammonium oxidation may promote *Z. taiwanensis* growth under anoxic conditions. In control experiments with nitrate-N (0.3, 3, or 30 mM) in the absence of ammonium, growth yields were lower (0.01 ± 0.00, 0.03 ± 0.02, and 0.09 ± 0.03, respectively), confirming negligible denitrification-driven growth in the absence of ammonium. Acetoxime and acetone were detected in all ammonium groups, with acetone production accounting for 5.2%–17.0% of consumed pyruvate. This proportion declined with increasing ammonium concentrations, implying that a lower C/N ratio may attenuate the diversion of pyruvate toward acetone. Additionally, in some tests, particularly those with 50 mM ammonium, residual ammonium was consistently observed. This persistence likely resulted from progressive acidification during ammonium oxidation [equations (4 and 8)], where the released H^+^ progressively lowered the pH, thereby inhibiting the ammonium oxidation process (pH values below 7.0 were found to significantly inhibit ammonium oxidation by *Z. taiwanensis* [11]).

### Synthesis and degradation of acetoxime during the ammonium-to-N_2_ conversion

**Figure 2:**
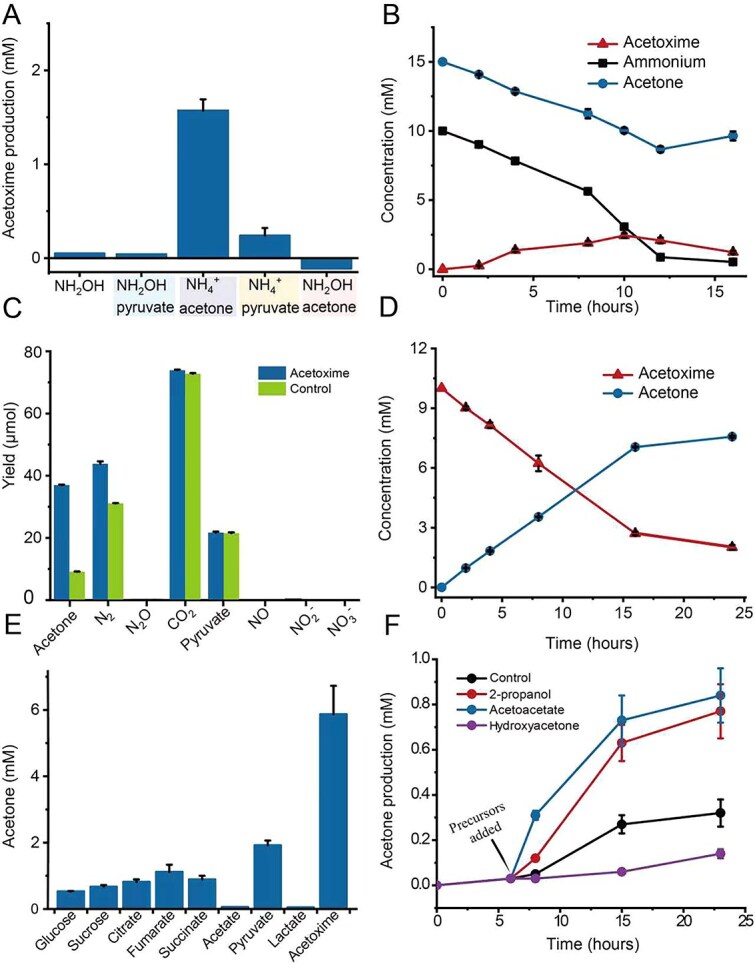
Involvement of acetoxime and acetone in the ammonium oxidation process by *Z. taiwanensis*. (A) Detection of substrates for acetoxime synthesis using harvested *Z. taiwanensis* cells. Certain combinations of 2 mM carbon sources and 2 mM nitrogen sources were used as substrates for acetoxime synthesis. Replications (B) were carried out using 10 mM ammonium and 15 mM acetone as substrates (no citrate). (C) Detection of decomposition products of acetoxime by *Z. taiwanensis* in BM. Specific carbon and nitrogen compounds were determined with or without the presence of 10 mM acetoxime after incubations in BM. (D) Replications to determine acetone as the product of acetoxime. Harvested cells were inoculated into a modified medium (without ammonium and citrate) containing 10 mM acetoxime. (E) Detection of substrates for acetone production. (F) Detection of the direct precursor for acetone production. 2 mM of acetoacetate, 2-propanol, and hydroxyacetone were added into a medium (no citrate) containing 1 mM of pyruvate at 6 h (the arrow), respectively. The culture without these added compounds was used as control. *n* = 3 (error bars, s.d.).

**Figure 3:**
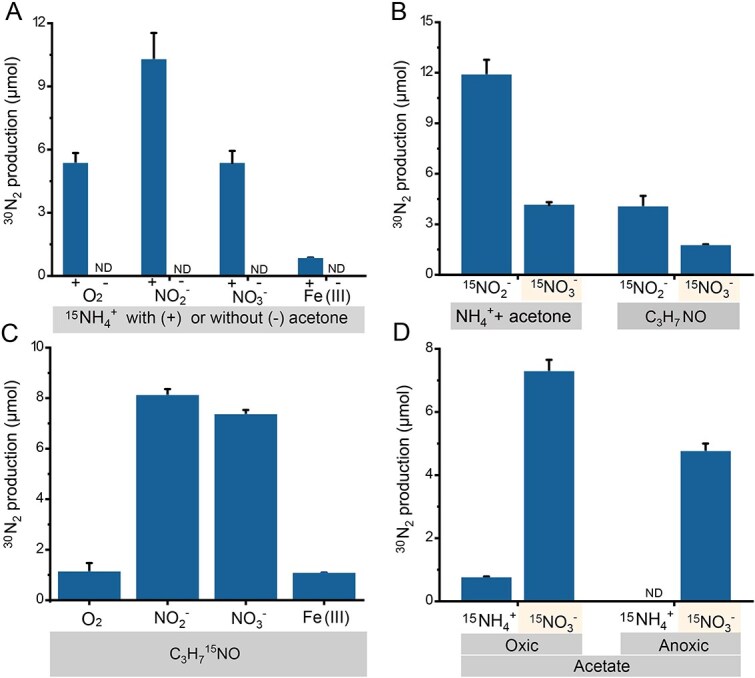
Determination of acetoxime as the intermediate and N_2_ as the end product of acetone-mediated ammonium oxidation using *Z. taiwanensis* cells. Harvested cells were inoculated into a modified medium devoid of any carbon and nitrogen sources. Carbon and nitrogen materials were subsequently added according to the following settings (A–D). (A) ^15^N-labeled ammonium with or without acetone was treated with O_2_ under oxic conditions (He/O_2_ = 1:1) and NO_2_^−^, NO_3_^−^, or Fe(III) under anoxic conditions (pure He). (B) Non-^15^N-labeled ammonium or acetoxime (C_3_H_7_NO) were treated with ^15^N-labeled NO_2_^−^ or NO_3_^−^. (C) ^15^N-labeled acetoxime was treated with O_2_ under oxic conditions and NO_2_^−^, NO_3_^−^, or Fe(III) under anoxic conditions. (D) ^15^N-labeled ammonium or nitrate was treated with acetate under oxic or anoxic conditions.

Acetoxime is known to be spontaneously synthesized from hydroxylamine and acetone through chemical reactions [32], but our data (Supplementary Fig. S1D, Supplementary Table S3) suggested that hydroxylamine is not involved in the biosynthesis of acetoxime (Supplementary information). Instead, *Z. taiwanensis* was capable of synthesizing a significant amount of acetoxime from acetone and ammonium (Fig. 2A), suggesting that ammonium and acetone might be direct precursors. To verify this conjecture, harvested cells were incubated in a modified medium containing acetone and ammonium as the sole carbon and nitrogen substrates (Fig. 2B). This condition resulted in a substantial accumulation of acetoxime (up to 25% ammonium), which decreased as most of the ammonium was consumed. This also indicated that *Z. taiwanensis* may further convert acetoxime. Furthermore, a decrease in pH (9.01 → 7.73) was observed, which can be attributed to the release of H^+^ during the oxidation of ammonium. To find the products of acetoxime oxidation, *Z. taiwanensis* was grown with ammonium, citrate, and acetoxime. Compared to the control without acetoxime addition, substantially higher quantities of acetone and N_2_ were observed, while there was no significant difference in other compounds such as pyruvate, CO_2_, NO_2_^−^, NO_3_^−^, NO, and N_2_O (Fig. 2C). This observation suggested that the oxidation products of acetoxime are predominantly acetone and N_2_. Subsequent replications showed that the amount of acetone released correlated stoichiometrically with the decrease in acetoxime (Fig. 2D), and the pH value remained constant. The production of N_2_ as the end product of ammonium oxidation was strongly supported by ^15^N labeling experiments (Fig. 3A, C, and  D). Regardless of whether ^15^N-labeled ammonium (in the presence of acetone) or acetoxime was used, both were oxidized to ^30^N_2_ under oxic conditions. The presence of N_2_, as the end product under anoxic conditions, was also verified through ^15^N-labeled experiments with the addition of NO_2_^−^, NO_3_^−^, or Fe(III) (Fig. 3A–C). When NO_2_^−^ or NO_3_^−^ was labeled with ^15^N in the presence of unlabeled ammonium or acetoxime, primarily, ^30^N_2_ was produced (Fig. 3B, Supplementary Fig. S4B), suggesting that complete denitrification occurred and there was no obvious nitrogen exchange between ammonium oxidation and denitrification. The highest N_2_ production rate was observed when nitrite was used as the electron acceptor. When *Z. taiwanensis* cells were incubated with identical concentrations of 10 mM ammonium, acetone, and nitrite, near-stoichiometric exhaustion of both ammonium and nitrite was observed (Supplementary Fig. S5).

### Role of acetone in the ammonium-to-N_2_ conversion

It was discovered that acetone serves as both a synthetic substrate and a decomposition product of acetoxime. Acetone appeared to play a mediator role in the conversion of ammonium to N_2_. As expected, the effective oxidation of ammonium by *Z. taiwanensis* ceased when acetone was absent in the cultures (Fig. 3A), indicating its crucial role in the ammonium oxidation process. The tests showed that *Z. taiwanensis* is capable of converting various organic compounds into acetone (Fig. 2E). To investigate the direct precursors for acetone production, acetoacetate, 2-propanol, and hydroxyacetone were added to cultures. Significant acetone production was observed in both the 2-propanol and acetoacetate groups (Fig. 2F), suggesting that the acetone production pathway in *Z. taiwanensis* is not singular and likely depends on the substrate utilized (Supplementary information). Acetone can also serve as a carbon source for the growth of certain microbes [45, 46], but our attempts to utilize acetone or acetoxime for *Z. taiwanensis* growth were unsuccessful. This indicates that *Z. taiwanensis* lacks pathways to metabolize acetone, leading to acetone accumulation (Fig. 1A). One role of acetone production lies in its function as a mediator for the ammonium-to-N_2_ conversion.

In conclusion, the pathway observed is an acetone-mediated ammonium oxidation process (ammonium + acetone → acetoxime → N_2_ + acetone, AMAO), which utilizes various electron acceptors, including O_2_, NO_2_^−^, NO_3_^−^, and Fe(III) [equations (1–10)].


(1) \begin{equation*} {{\mathrm{NH}}_4}^{+}+{\left({\mathrm{CH}}_3\right)}_2\mathrm{C}=\mathrm{O}\to{\left({\mathrm{CH}}_3\right)}_2\mathrm{C}=\mathrm{N}-\mathrm{OH}+2\mathrm{e}-+{3\mathrm{H}}^{+} \end{equation*}

$$ {\Delta \mathrm{G}}^{0^{\prime}}=+382.0\ \mathrm{kJ}\ {\mathrm{mol}}^{-1} $$

(2) \begin{equation*} {\left({\mathrm{CH}}_3\right)}_2\mathrm{C}=\mathrm{N}-\mathrm{OH}\to 0.{5\mathrm{N}}_2+{\left({\mathrm{CH}}_3\right)}_2\mathrm{C}=\mathrm{O}+\mathrm{e}-+{\mathrm{H}}^{+} \end{equation*}

$$ {\Delta \mathrm{G}}^{0^{\prime}}=-302.7\ \mathrm{kJ}\ {\mathrm{mol}}^{-1} $$

With O_2_ as the electron acceptor:


(3) \begin{equation*} {3\mathrm{H}}^{+}+3\mathrm{e}-+0.75{\mathrm{O}}_2\to 1.{5\mathrm{H}}_2\mathrm{O} \end{equation*}

$$ {\Delta \mathrm{G}}^{0^{\prime}}=-355.7\ \mathrm{kJ}\ {\mathrm{mol}}^{-1} $$

(4) \begin{equation*} {{\mathrm{NH}}_4}^{+}+0.75{\mathrm{O}}_2\to 0.{5\mathrm{N}}_2+1.{5\mathrm{H}}_2\mathrm{O}+{\mathrm{H}}^{+} \end{equation*}

$$ {\Delta \mathrm{G}}^{0^{\prime}}=-276.4\ \mathrm{kJ}\ {\mathrm{mol}}^{-1} $$

With NO_2_^−^ as the electron acceptor:


(5) \begin{equation*} {4\mathrm{H}}^{+}+3\mathrm{e}-+{\mathrm{NO}}_2-\to{2\mathrm{H}}_2\mathrm{O}+0.{5\mathrm{N}}_2 \end{equation*}

$$ {\Delta \mathrm{G}}^{0^{\prime}}=-442.0\ \mathrm{kJ}\ {\mathrm{mol}}^{-1} $$

(6) \begin{equation*} {{\mathrm{N}\mathrm{H}}_4}^{+}+{\mathrm{N}\mathrm{O}}_2-\to{\mathrm{N}}_2+{2\mathrm{H}}_2\mathrm{O} \end{equation*}

$$ {\Delta \mathrm{G}}^{0^{\prime}}=-362.7\ \mathrm{kJ}\ {\mathrm{mol}}^{-1} $$

With NO_3_^-^ as the electron acceptor:


(7) \begin{equation*} {6\mathrm{H}}^{+}+5\mathrm{e}-+{\mathrm{NO}}_3-\to{3\mathrm{H}}_2\mathrm{O}+0.{5\mathrm{N}}_2 \end{equation*}

$$ {\Delta \mathrm{G}}^{0^{\prime}}=-600.0\ \mathrm{kJ}\ {\mathrm{mol}}^{-1} $$

(8) \begin{equation*} {{\mathrm{NH}}_4}^{+}+0.{6\mathrm{NO}}_3-\to 0.{8\mathrm{N}}_2+0.{4\mathrm{H}}^{+}+1.{8\mathrm{H}}_2\mathrm{O} \end{equation*}

$$ {\Delta \mathrm{G}}^{0^{\prime}}=-280.7\ \mathrm{kJ}\ {\mathrm{mol}}^{-1} $$

With Fe(III) as the electron acceptor:


(9) \begin{equation*} \mathrm{e}-+{\mathrm{Fe}}^{3+}\to{\mathrm{Fe}}^{2+} \end{equation*}

$$ {\Delta \mathrm{G}}^{0^{\prime}}=-280.7\ \mathrm{kJ}\ {\mathrm{mol}}^{-1} $$

(10) \begin{equation*} {{\mathrm{NH}}_4}^{+}+{3\mathrm{Fe}}^{3+}\to 0.{5\mathrm{N}}_2+{4\mathrm{H}}^{+}+{3\mathrm{Fe}}^{2+} \end{equation*}

$$ {\Delta \mathrm{G}}^{0^{\prime}}=-143.3\ \mathrm{kJ}\ {\mathrm{mol}}^{-1} $$

### Key enzymes for AMAO

**Figure 4:**
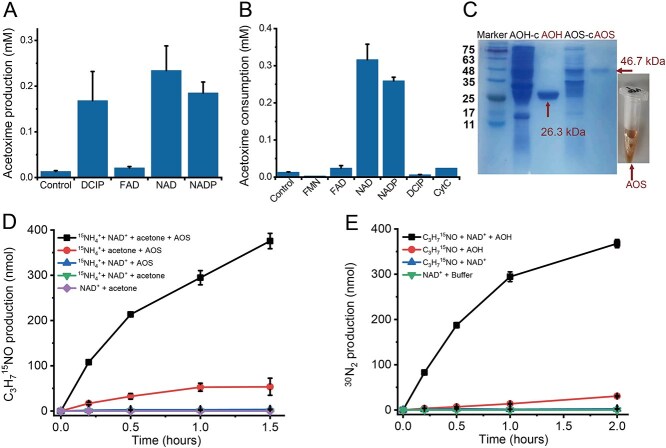
Purification and characterization of AOS and AOH from *Z. taiwanensis*. (A, B) The activity of AOS (A) and AOH (B) was tested with various electron acceptors. (C) SDS-PAGE analysis of AOS and AOH. AOH-c and AOS-c, indicating crude AOH and AOS enzymes, respectively. (D) Production of ^15^N-labeled acetoxime by purified acetoxime synthase (AOS). C_3_H_7_^15^NO was produced with the highest rate when AOS (5.6 μg) was incubated 2 mM ^15^NH_4_Cl, 3 mM acetone, and 6 mM NAD^+^. (E) Production of ^15^N_2_ by purified acetoxime dehydrogenase (AOH). ^15^N_2_ was produced with the highest rate when AOH (3.7 μg) was incubated with 2 mM C_3_H_7_^15^NO and 5 mM NAD^+^. ND means not detected. Control experiments were incubated under the same experimental conditions; *n* = 3 (error bars, s.d.).

According to the AMAO pathway described above, two enzymes would be needed: AOS and AOH. The AOS enzyme was extracted from the cell membrane using dodecyl β-D-maltoside and subsequently purified using anion exchange and gel filtration chromatography. In contrast, AOH was purified from the supernatant of the cell-free extract using two types of anion exchange, followed by gel filtration chromatography. Both enzymes were found to utilize NAD^+^/NADP^+^ as electron acceptors (Fig. 4A and B). Visually, the purified AOS was brown and exhibited a molecular weight of ~47 kDa as characterized by sodium dodecyl sulfate–polyacrylamide gel electrophoresis (SDS-PAGE) analysis (Fig. 4C). The purified AOH was ~26 kDa and appeared colorless (Fig. 4C). AOS showed significant activity not only with NAD^+^/NADP^+^ but also in the presence DCIP, suggesting potential diversity in the electron acceptors utilized by AOS. Considering its brown color, the AOS is strikingly similar to typically FAD-dependent enzymes with FAD being a bound cofactor [47]. In contrast, AOH exhibited high activity exclusively in the presence of NAD^+^/NADP^+^. The ^15^N labeling experiment results (Fig. 4D and E) indicated that the purified AOS efficiently catalyzed the formation of acetoxime (376 ± 17 nmol) after ammonium (388 ± 11 nmol) and acetone (429 ± 6 nmol) consumption in the presence of NAD^+^, whereas purified AOH facilitated the oxidation of acetoxime (751 ± 19 nmol) to N_2_ (368 ± 9 nmol) and acetone (730 ± 10 nmol), also in the presence of NAD^+^, under anoxic conditions. This provides additional strong evidence supporting the proposed AMAO pathway (ammonium + acetone → acetoxime → N_2_ + acetone). The generation of NADH suggested that *Z. taiwanensis* may obtain energy through the AMAO process. To evaluate the interaction between AMAO enzymes and the respiratory nitrite reductase, nitrate reductase, and ferric reductase, we prepared a cell-free extract of *Z. taiwanensis* containing NAD^+^-linked enzymes (AOS and AOH), reductases (nitrite reductase, nitrate reductase, and ferric reductase) [48–51], and NAD^+^/NADH-linked materials (the quinone pool, ferredoxin, and cytochrome c, as well as enzymes that facilitate the transfer of electrons between these compounds and NAD^+^/NADH). Experiments revealed that adding either NAD^+^ or NADH drove the simultaneous depletion of ammonium and electron acceptors (NO_2_^−^, NO_3_^−^, or Fe^3+^) (Supplementary Fig. S6). This coupling occurs through a redox cycle in which NADH produced by AOS/AOH [equations (11 and 12)] enhances the reduction of NO_2_^−^, NO_3_^−^, or Fe^3+^, while the resulting NAD^+^ reactivates AOS and AOH.


(11) \begin{equation*} {{\mathrm{NH}}_4}^{+}+{\left({\mathrm{CH}}_3\right)}_2\mathrm{C}=\mathrm{O}+{\mathrm{NAD}}^{+}\to{\left({\mathrm{CH}}_3\right)}_2\mathrm{C}=\mathrm{N}-\mathrm{OH}+\mathrm{NADH}+{2\mathrm{H}}^{+} \end{equation*}

(12) \begin{align*} &{\left({\mathrm{CH}}_3\right)}_2\mathrm{C}=\mathrm{N}-\mathrm{OH}+0.{5\mathrm{NAD}}^{+}\to 0.5\ {\mathrm{N}}_2+{\left({\mathrm{CH}}_3\right)}_2\mathrm{C}\nonumber \\ &\qquad =\mathrm{O}+0.5\mathrm{NAD}\mathrm{H}+0.{5\mathrm{H}}^{+} \end{align*}

### Gene sequences encoding for acetoxime synthase and acetoxime dehydrogenase enzymes and their distribution

**Figure 5:**
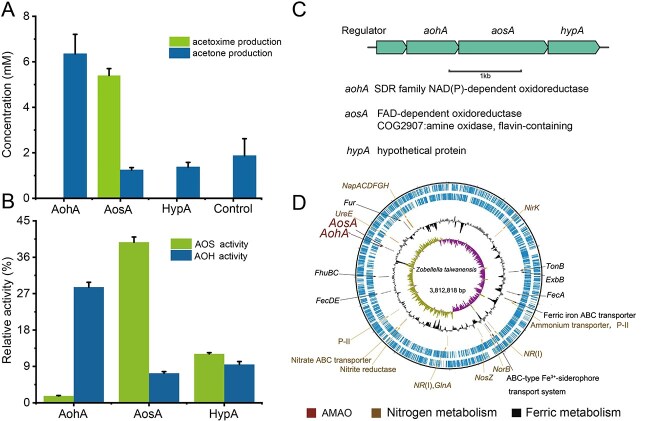
Characterization of *aosA* and *aohA* genes. (A) Demonstration of acetoxime and acetone production in *E. coli* harboring genes *aohA, aosA,* and *hypA*. *E. coli* DH5α with an empty pBAD-HisA plasmid served as the control. (B) Enzyme activity of AOS and AOH expressed by *E. coli* DH5α (relative to the purified enzymes from *Z. taiwanensis*); *n* = 3 (error bars, s.d.). (C) The gene cluster with potential genes encoding for AOS and AOH enzymes and their gene annotation. (D) The circular genome map of *Z. taiwanensis*. Circles from outermost to center show: Protein-coding sequences (CDS; rings 1, 2); genes of enzymes involved in ferric metabolism, AMAO, and other pathways of catabolic nitrogen metabolism (rings 3); GC content (rings 4, 5); GC skew (rings 6, 7). A list of gene names and corresponding enzymes is provided in Supplementary Table S6.

The genome of *Z. taiwanensis* DN-7 contains a chromosome of 3 812 818 bp (Supplementary Table S4). Acetone carboxylase [46] was absent, confirming *Z. taiwanensis* cannot utilize acetone as a carbon source for growth. To screen the AOS and AOH genes, a fosmid library of *Z. taiwanensis* containing 952 clones was constructed successfully, which reached 99% sequence coverage of the genome. We developed a selective medium (glucose-C/ammonium-N = 0.1) to enrich for clones utilizing the abundant ammonium for rapid growth via AMAO. This approach identified a potential clone, AC3, which exhibited both AOS (14.3 ± 3.6 U mg^−1^) and AOH (16.5 ± 2.2 U mg^−1^) activity. The inserted fragment size of the AC3 fosmid was 42 kb (Supplementary Table S5), which contains two potential genes (*aosA* and *aohA*) coding for AOS and AOH (Fig. 5C). The AosA protein with 47 kDa belongs to the Zeta Carotene Desaturase and Related Oxidoreductases family and has two predicted functions: FAD-dependent oxidoreductase or amine oxidase, flavin containment (Supplementary Fig. S7A). This prediction was consistent with characterized AOS enzymes, which were known to contain an essential FAD cofactor and catalyze ammonium oxidation reactions. In addition, the predicted function of the AohA protein with 26 kDa is a Rossmann-fold NAD (P)-dependent oxidoreductase belonging to the SDR family (Supplementary Fig. S7B), aligning with the known data for AOH. To clarify the specific functions of these candidates, we cloned them onto the pBAD-HisA plasmid individually and transferred them into *E. coli* DH5α (without similar sequences to *aosA* and *aohA*) for heterologous expression. Only the *aohA* clone showed significant AOH activity (with maximal acetoxime removal), while only the *aosA* clone exhibited apparent AOS activity (with acetoxime accumulation) (Fig. 5A and B). Moreover, LCMS analysis of the purified enzymes using the AosA and AohA protein sequences as references confirmed that the AohA protein was indeed AOH with 26.3 kDa and the AosA protein was identified as AOS with 46.7 kDa. These molecular weights were consistent with the ones observed in SDS-PAGE analysis (Fig. 4C).

Gene *aosA* (1248 bp) encoded a 415-aa AOS protein (pI 7.03), while *aohA* (732 bp) encoded a 243-aa AOH (pI 9.79). BLASTP searches detected the presence of homologous proteins in thousands of prokaryotic and eukaryotic genomes (Supplementary information, Fig. 6A and B), suggesting that AMAO may be widespread in nature. It is still unknown if all these homologous protein sequences truly perform AOS and AOH, but at least a group of sequences from many *Gammaproteobacteria* were in the same evolutionary clade as *Z. taiwanensis* AOS/AOH (Supplementary Fig. S8A and B). We found that most reported heterotrophic bacteria capable of ammonium-to-N_2_ conversion (*Aeromonas* sp*.*, *K. pneumoniae*, *Providencia rettgeri*, *Serratia marcescens*, *V. diabolicus*, *Photobacterium* sp*.*, *Halomonas* sp*.*, *Pseudomonas putida*, *Agrobacterium* sp., *Alcaligenes faecalis*, *Cupriavidus* sp*.*, *Diaphorobacter* sp*.*, *Acinetobacter* sp*.*, *Arthrobacter* sp*.*, *Microbacterium* sp*.*, *Rhodococcus* sp*.*, *B. subtilis*, and *Exiguobacterium* sp*.*) [12–15, 18–24, 52–58] and nitrogen-removing fungi (*Pichia kudriavzevii*, *Fusarium* sp*.*, and *Aspergillus* sp*.*) [59–61] have sequences similar to AOS and AOH, suggesting potential AMAO capacity in these microbes.

**Figure 6:**
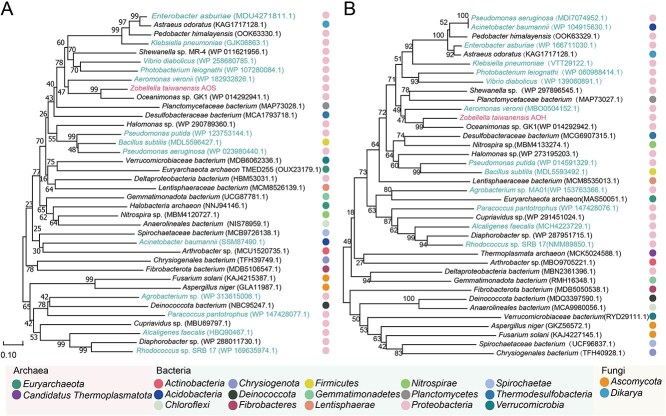
Phylogeny of AOS and AOH from *Z. taiwanensis* and related proteins. Neighbor-joining trees showing AOS (A) and AOH (B) phylogenies. The scale bars represent 0.1 estimated substitutions per residue. Taxa referring to the species known for their ability to convert ammonium to N_2_ are highlighted. Proteins associated with various microbial taxa at the phylum level are marked with different-colored circles.

## Discussion

**Figure 7:**
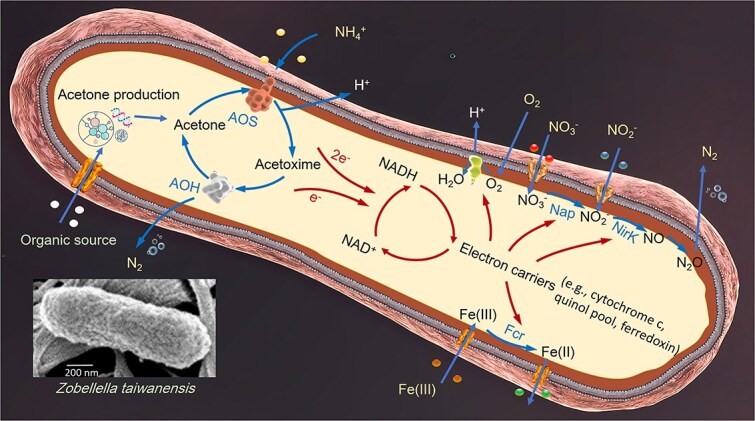
Biochemical pathway of AMAO in *Z. taiwanensis*. Scanning electron micrograph of *Z. taiwanensis* is shown in the bottom left side of the figure. Arrows depict the AMAO pathway, its coupling to the reduction of O_2_, NO_2_^−^, NO_3_^−^, and Fe(III), and the associated electron flow; AOS, acetoxime synthase; AOH, acetoxime dehydrogenase; Nap, periplasmic nitrate reductase; NirK, copper containing nitrite reductase; Fcr, ferric reductase.

Based on the experimental findings, we present a biochemical pathway of AMAO (Fig. 7). To our knowledge, AMAO represents a unique mechanism. The biosynthesis of acetone from organic matter is a well-documented phenomenon, with numerous studies highlighting its occurrence in both natural and engineered ecosystems [62–64]. Although the ecological significance of acetone production by heterotrophs remains unclear, we hypothesize that one of its functions is to mediate an ammonium-to-N_2_ oxidation. In *Z. taiwanensis*, acetone production rates varied significantly (5.2%–20.4%) depending on carbon source and C/N ratios. The variability in acetone production likely fulfills other roles [46, 65], including diverting excess carbon to acetone, which may alleviate metabolic bottlenecks, mitigating equivalent accumulation of carbon intermediates, and potentially suppressing competing microorganisms.

Acetoxime readily undergoes oxidation to yield acetone and N_2_ [32]. Drawing parallels to the known catalytic mechanisms of enzymes responsible for N-N bond formation [66], we propose that the AOH enzyme may follow a similar process. Specifically, upon binding to the active site of AOH enzymes, acetoxime may undergo hydrolysis to release acetone and hydroxylamine. The N-OH bond of hydroxylamine could then be cleaved to generate a nitrogen radical (•N=), which dimerizes to form an N=N intermediate with another nitrogen radical. This intermediate is further oxidized to form an N ≡ N triple bond, ultimately releasing N_2_. The lost electrons are transferred to NAD^+^ bound to the AOH enzyme.

Although ammonium oxidation by heterotrophs was commonly thought to be energetically costly [5, 25], *Z. taiwanensis* demonstrates respiratory characteristics in AMAO through NADH generation and multi-acceptor electron transfer. However, in oxic pyruvate cultures, the growth contribution of AMAO was not observed. This is likely because under oxic conditions, the bacterium preferentially utilizes readily oxidizable pyruvate as an energy source, whereas under anoxic conditions, pyruvate may not be fully oxidized, making the energy yield from AMAO more significant. Future studies employing more recalcitrant carbon sources could better elucidate the potential cellular energy conservation of AMAO.

Despite uncertain energetic contributions, AMAO provides a unique competitive advantage via exceptional electron acceptor versatility. Unlike the known microbial ammonium oxidation, AMAO represents a newly reported instance of both aerobic and anaerobic ammonium oxidation, expanding the traditional notion that heterotrophic ammonium oxidation occurs exclusively under strict oxic conditions [5, 25]. Its ability to utilize ubiquitous electron acceptors (O_2_, NO_3_^−^, and Fe^3+^) enhances adaptation across oxygen gradients in natural and engineered ecosystems. We further hypothesize that additional acceptors like Mn^4+^ may also support AMAO.

Environmental competitive advantage of AMAO likely promotes the widespread distribution of microorganisms carrying this pathway. Emerging evidence predicts a new ammonium-to-N_2_ pathway in oxic riverbeds [67] and highlighted the significant role of heterotrophs in anoxic sediments [27]. The shared sequences of AOS and AOH among heterotrophic bacteria, such as those from *Proteobacteria*, *Acidobacteria*, *Actinobacteria*, and *Firmicutes*, which were dominant phyla in many soils [68], sediments [69], and sewage treatment facilities [70], suggests potential broad environmental occurrence of AMAO. Recent reports have explored the relationship between ammonium and acetone in terrestrial soil, showing that acetone application can inhibit nitrification but enhance total dissolved nitrogen removal [71]. Another study demonstrated a significant increase in acetone release after introducing NH_4_NO_3_ to soil [72].

In summary, this study unveils a new pathway for ammonium-to-N_2_ conversion expanding our understanding of ammonium oxidation by heterotrophs, illuminating a new intricate interplay between carbon and nitrogen metabolism in microbial ecosystems. Further study is warranted to explore the exact role of this pathway in the nitrogen cycle.

## Supplementary Material

Supplementary_information_wraf230

## Data Availability

Genomic sequence data of *Z. taiwanensis* in this study have been deposited at NCBI with sequence accession number CP154835. The nucleotide and amino acid sequences of the AOS and AOH have been assigned the GenBank accession number (PP694646, PP694647).
