# Supplementary material for: Acetone-mediated ammonium oxidation to dinitrogen by Zobellella taiwanensis bacteria
Source: ISME J. 2025 Oct 27;19(1):wraf230. doi: 10.1093/ismejo/wraf230 (PMC12596615; doi:10.1093/ismejo/wraf230)
Supplement: Supplementary_information_wraf230 [file supplementary_information_wraf230.pdf]

Supplementary information

Acetone-mediated ammonium oxidation to dinitrogen by *Zobellella taiwanensis* bacteria

Authors: Yu Lei[1, 2, 3], Yangqing Wang[1, 2, 3], Xiaojuan Tan[4], Chuanwu Xi[5, *], Hong Liu[1, 2, *]

Affiliations:

[1]Chongqing Institute of Green and Intelligent Technology, Chinese Academy of Sciences, Chongqing, 400714, China

[2]Chongqing School, University of Chinese Academy of Sciences, College of Resources and Environment, Chongqing, 400714, China

[3]Chongqing University, Chongqing, 400044, China

[4]Anhui Provincial Key Laboratory of Molecular Enzymology and Mechanism of Major Metabolic Diseases, College of Life Sciences, Anhui Normal University, Wuhu, 241000, China

[5]Department of Environmental Health Sciences, University of Michigan, Ann Arbor, MI, 48109, USA

[*]**Corresponding author:** No. 266 Fangzhen Avenue, Shuitu High-Tech Park, Beibei, Chongqing 400714, China.

E-mail address: H.L. liuhong@cigit.ac.cn; C. X. cxi@umich.edu

Supplementary Methods

Inhibitor experiments

To investigate the impact of sodium diethyldithiocarbamate (DDC) on ammonium oxidation, *Z. taiwanensis* were subjected to treatment with and without DDC. A 1% *Z. taiwanensis* culture was inoculated into 350 ml triangular flasks containing 100 ml of BM with or without 0.1 mM DDC. The flasks were then incubated at 30 ℃ and 150 rpm. NH_4^+, NO_2^- and NO_3^- were measured after 12, 24, 48, and 72 hours of incubation. To assess the influence of DDC on acetoxime oxidation during ammonium oxidation by *Z. taiwanensis*, acetoxime ranged from 0.1 to 5.0 mM were introduced into the BM, respectively. Following inoculation and incubation as described above, samples (500 μl) for NO_2^- determination were taken immediately after 6, 16, 24, and 40 hours of incubation. Additionally, the utilization of hydroxylamine was tested by inoculating 1% cultures in BM containing hydroxylamine hydrochloride as the sole nitrogen source instead of ammonium. The cultures were incubated at 30 ℃ and 150 rpm for 96 hours. The growth OD_{600}, hydroxylamine and nitrite were measured.

To assess the effect of acetylene on ammonium oxidation, *Z. taiwanensis* were treated to with and without acetylene. Tightly sealed bottles, totaling 340 ml in volume, were filled with 50 ml of BM and inoculated with overnight culture of the *Z. taiwanensis*. To ensure oxic conditions, the top air of the sealed bottles was removed by flushing the bottles with 1:1 of He/O_2 (99.99% purity) flushing for a duration of 20 minutes. Then bottles were injected with acetylene (yielding a final concentration of 5%) and incubated at 30 ℃ at 150 rpm. Prior to injection, the acetylene (>98% purity) was purified by flushing through a tube of concentrated H_2SO_4 and subsequent flushing through distilled water. Gas samples (500 μl) were taken from the headspace by a 1 ml gas-tight syringe (SGE, Australia) after 12, 24, 48, and 72 hours incubation, and N_2 and N_2O concentration were determined. To evaluate the predicted intermediates involved in the ammonium oxidation process, ^{15}N labeled compounds were introduced to the BM containing 5 mM $(NH_4)_2SO_4$. *Z. taiwanensis* was incubated with or without acetylene as mentioned above. After 6 hours of incubation, specific predicted intermediates were introduced into the cultures. This involved adding ^{15}N-labeled NH_2OH, NO_2^-, NO, N_2O, or acetoxime to achieve final concentrations of 1 mM or 0.05 mM as appropriate. As a control, several bottles consisted of 5mM ^{15}N-labeled $(NH_4)_2SO_4$ with or without 5% acetylene were incubated without addition of the predicted intermediates. The initial abundance of ^{15}N was adjusted to 10% by mix non-labeled and labeled materials. The bottles without *Z. taiwanensis* inoculation were also used as control. After incubation, the concentration and the ^{15}N abundance of N_2O in the headspace were detected.

Genome sequencing of *Z. taiwanensis*

Z. taiwanensis cells in late-log growth phase were harvested, and genomic DNA was extracted using Wizard Genomic DNA purification kit (Promega, USA). The quality and quantity of the obtained genomic DNA were determined using 0.8% agarose gel electrophoresis and Nanodrop ND-1000 Spectrophotometer (Thermo Fisher Scientific). Subsequently, 30 μg of DNA was used for sequencing via PacBio single-molecule real-time (SMART) technology at University of Michigan. The reads were *de novo* assembled by hierarchical genome assembly process in SMRT analysis V2.3.0, and the cleaned sequence was submitted to Pacbio portal’s Resequencing protocol for polishing the assembled sequence. The overlapping regions between the two ends of the assembled sequence were identified, and one end was trimmed to circularize the sequence. The open reading frame (ORF) prediction and annotation were done by Glimmer 3. Structural rRNAs and tRNAs were determined using RNAmmer 1.2 and tRNAscan 1.31, respectively. In addition, automated genome annotation was performed using Rapid Annotation using Subsystem Technology (RAST)

serve. A COG analysis of the RAST annotated protein sequences was performed on the WebMGA server, which carry out functional annotation using the RPSBLAST program on the COG database.

LCMS

The purified AOS and AOH were digested with trypsin, desalinated using C18 desalinating column and freeze-dried for sample preparation. Prepare mobile phase A (100% water, 0.1% formic acid) and mobile phase B (80% acetonitrile, 0.1% formic acid). Dissolve lyophilized powder in 10 μL of mobile phase A, centrifuge at 14000 g for 20 minutes at 4°C, and take 1 μg of the supernatant for injection and liquid chromatography-mass spectrometry analysis. A Q Exactive HF-X mass spectrometer with a Nanospray Flex (NSI) ion source is used. The ion spray voltage is set to 2.2 kV, and the ion transfer tube temperature is 320 ℃. Mass spectrometry is performed in a data-dependent acquisition mode with a full scan range of m/z 350-1500. The resolution of the first mass spectrometry scan is set to 120,000 (at 200 m/z), with an automatic gain control (AGC) target of 3×10^6 and a maximum injection time of 80 ms. The top 40 ions based on intensity in the full scan are selected for high-energy collision dissociation fragmentation in the second mass spectrometry scan. The resolution of the second mass spectrometry scan is set to 15,000 (at 200 m/z) with an AGC target of 5×10^4 and a maximum injection time of 45 ms. The collision energy for peptide fragmentation is set at 27%. The acquired mass spectrometry raw data was searched against a database of predicted acammox sequences from positive pCC1FOS clones obtained earlier.

3D modeling for the predicted structures of AOS and AOH

The complete amino acid sequences of each protein were inputted into the structure prediction module in the AlphaFold Protein Structure Database (https://alphafold.ebi.ac.uk/). For each sequence submission, the full predicted atomic coordinates were retrieved in PDB format, including the associated per-residue confidence scores (pLDDT). These structures were then cross-referenced with the InterPro database (https://www.ebi.ac.uk/interpro/) by submitting the same protein sequences through their sequence search tool to identify conserved domains and structural features. All structural visualizations were prepared using PyMOL (v2.5.2).

Sequence collection and phylogenetics.

For phylogenetic analyses of AOS and AOH, amino acid sequences and gene sequences were downloaded from the NCBI database. BLASTP/N searches were performed using the *Z. taiwanensis* AOS and AOH sequence as a query respectively, word size = 5, BLOSUM 62, E = 0.05 and the top 1,000 returned sequences were displayed. Selected sequences were aligned by ClustalW in MEGA 11, and neighbor-joining trees were constructed using MEGA 11.

Measurement of $^{15}N_2$ and $^{15}N_2O$ isotopic composition

The isotopic analysis of $^{15}N_2$ was performed using a GasBench II system coupled to a MAT 253 Plus isotope ratio mass spectrometer (Thermo Fisher Scientific). According to the Thermo Fisher Scientific technical manuals, gas samples containing $^{15}N_2$ were injected into 12 ml Labco exetainer vials that had been previously flushed with high-purity He for 20 minutes to remove atmospheric contamination. The automated sampling system (CTC PAL autosampler) introduced samples sequentially from a 96-position carousel into the GasBench II interface. Separation of N_2 was achieved using a Poraplot Q chromatographic column maintained at constant temperature, with high-purity He as the carrier gas. The eluted N_2 was introduced into the IRMS for measurement of ion currents at m/z 28 ($^{14}N_2$), 29 ($^{15}N^{14}N$), and 30 ($^{15}N_2$). The reference gas employed for continuous calibration throughout sample analysis was high-purity N_2 (99.99%). Instrument calibration was performed using the solid reference materials IAEA-N1 and USGS25. The concentrations of $^{29}N_2$ and $^{30}N_2$ in each sample were calculated using the formula: $C_{\text{iso}} = S_{\text{iso}} \times a^{-1} \times V_{\text{gas}}^{-1}$, where C_{iso} represents the concentration of $^{29}N_2$ or $^{30}N_2$, S_{iso} is the signal intensity of $^{29}N_2$ or

$^{30}N_2$, *a* denotes the linear calibration factor (signal/mole N_2) of the mass spectrometer, and V_{gas} refers to the volume of the injected gas sample in the 12-ml exetainer. The microbially produced $^{29}N_2$ or $^{30}N_2$ was determined by subtracting the values measured in no-bacteria controls from those in the sample vials.

For analysis of ^{15}N abundance in N_2O, a gas chromatograph-isotope ratio mass spectrometry system (Delta V Plus IRMS, Thermo Fisher Scientific) was employed. Gas samples (0.5 ml) were manually injected into the GasBench II interface using gastight syringes and passed through chemical traps (Ascarite) and a liquid nitrogen cold trap (-196 ℃) to remove CO_2 and water vapor while concentrating N_2O. The purified N_2O was separated on a Poraplot Q GC column before introduction into the IRMS. The instrument simultaneously monitored ion currents at m/z 44, 45, and 46 to determine δ15N values.

GCMS for $C_3H_7{}^{15}NO$ measurement

$C_3H_7{}^{15}NO$ was extracted from the mixture using dichloromethane and analyzed using a gas chromatograph-mass spectrometer (7890A-5977A, Agilent, USA) equipped with an HP-5MS fused-silica capillary column (30 m × 0.25 mm × 25 μm, Agilent). The carrier gas was helium with flow rate of 1.0 ml/min. Split ratio of 30:1 was used. Temperature program: 60°C → 10°C/min → 250°C → 20°C/min → 300°C (1 min). Total ion current was monitored for all samples using electron-impact ionization (70 eV). A scan rate of 1 scan/s was applied. Quantification was performed using characteristic mass.

Supplementary Text

Thermodynamic feasibility of the ammonium oxidation reactions by *Z. taiwanensis*

To evaluate the thermodynamic feasibility of the proposed equations, the $\Delta G^{0'}$ value was calculated. The $\Delta_f G^0$ value of NH_4^+ (-79.3 kJ mol^{-1}), NO_2^- (-32.2 kJ mol^{-1}), NO_3^- (-111.3 kJ mol^{-1}), Fe^{3+} (-4.7 kJ mol^{-1}), Fe^{2+} (-78.9 kJ mol^{-1}), H_2O(l) (-237.1 kJ mol^{-1}), acetone(l) (-147.7 kJ mol^{-1}), was obtained by searching the 97th CRC Handbook of Chemistry and Physics [1]. The $\Delta_f G^0$ value of acetoxime ($\Delta fG^{0'} \approx +155$ kJ mol^{-1}) was estimated. The $\Delta G^{0'}$ values of the equations (1)-(10) downside were caculated according to the formula: $\Delta G^{0'} = \sum \Delta_f G^0$ (product)-$\sum \Delta_f G^0$ (substrate).

$NH_4^+ + (CH_3)_2C{=}O \rightarrow (CH_3)_2C{=}N\text{-}OH + 2e^- + 3H^+$ (1)

$\Delta G^{0'} = [155 + (3\times 0)] – [(-79.3) + (-147.7)] = +382.0$ kJ mol^{-1}

$(CH_3)_2C{=}N\text{-}OH \rightarrow 0.5N_2 + (CH_3)_2C{=}O + e^- + H^+$ (2)

$\Delta G^{0'} = [(0.5\times 0) + (-147.7) + 0 + 0] – [155] = -302.7$ kJ mol^{-1}

$3H^+ + 3e^- + 0.75O_2 \rightarrow 1.5H_2O$ (3)

$\Delta G^{0'} = 1.5\times(-237.1) – [0 + 0 + 0] = -355.65$ kJ mol^{-1}

$NH_4^+ + 0.75O_2 \rightarrow 0.5N_2 + 1.5H_2O + H^+$ (4)

Sum of (1) + (2) + (3): $\Delta G^{0'} = -276.35$ kJ mol^{-1}

$4H^+ + 3e^- + NO_2^- \rightarrow 2H_2O + 0.5N_2$ (5)

$\Delta G^{0'} = [2\times(-237.1) + 0] – [0 + 0 + (-32.2)] = -442.0$ kJ mol^{-1}

$NH_4^+ + NO_2^- \rightarrow N_2 + 2H_2O$ (6)

Sum of (1) + (2) + (5): $\Delta G^{0'} = -362.7$ kJ mol-1

$6H^+ + 5e^- + NO_3^- \rightarrow 3H_2O + 0.5N_2$ (7)

$\Delta G^{0'} = [3\times(-237.1) + 0] – [0 + 0 + (-111.3)] = -600.0$ kJ mol^{-1}

$NH_4^+ + 0.6NO_3^- \rightarrow 0.8N_2 + 0.4H^+ + 1.8H_2O$ (8)

Sum of (1) + (2) + 0.6×(7): $\Delta G^{0'} = -280.7$ kJ mol^{-1}

$e^- + Fe^{3+} \rightarrow Fe^{2+}$ (9)

$\Delta G^{0'} = (-78.9) – (-4.7) = -74.2$ kJ mol^{-1}

$NH_4^+ + 3Fe^{3+} \rightarrow 0.5N_2 + 4H^+ + 3Fe^{2+}$ (10)

Sum of (1) + (2) + 3×(9): $\Delta G^{0'}$ = -143.3 kJ mol^{-1}

Contribution of nitrification and denitrification process to the conversion of ammonium to N_2 by *Z. taiwanensis*

To investigate the pathway for ammonium oxidation, sodium diethyldithiocarbamate (DDC), a Cu chelator [2], was utilized to inhibit the copper-containing nitrite reductase (NirK) during the ammonium oxidation process by *Z. taiwanensis* (Supplementary Fig. 1A). However, the ammonium oxidation was not impeded, and the addition of DDC led to a slight higher accumulation of nitrite (NO_2^--N < 1% of initial ammonium-N), whereas the absence of DDC resulted in minimal nitrite accumulation under oxic conditions. This implies that the nitrite produced from ammonium could be rapidly and aerobically denitrified by *Z. taiwanensis*, possibly due to the presence of NirK. Nitrate was not observed in either scenario with or without DDC. It is postulated that only partial nitrification occurs, or the high activity of nitrate reductase hinders nitrate accumulation. The DDC results indicated that less than 1% of ammonium was converted to nitrite. Additionally, another inhibitor, acetylene, was unable to halt the ammonium oxidation process (Supplementary Fig. 1B), distinguishing it from autotrophic nitrifying microbes that contains AMO [3, 4]. It was observed that acetylene could inhibit N_2O reduction [4], but the accumulation of N_2O was also minimal (N_2O-N < 1% of initial ammonium-N).

To confirm that N_2O is produced from ammonium via nitrification and denitrification process, we repeated acetylene experiments with ^{15}N-labeled ammonium (10% ^{15}N abundance, not pure ^{15}N) as the sole nitrogen source. We found that when acetylene was not added, no labeled N_2O was detected, only after adding acetylene was labeled N_2O detected, but the accumulated $^{15}N_2O$-N was also very low, less than 1% of the initial NH_4^+-N (Supplementary Table 3). This result indicates that less than 1% of ammonium was converted to N_2O through nitrification and denitrification process.

To confirm the potential intermediate, we added 1 mM of labeled N-containing additives (with 10% ^{15}N abundance, not 99%) to the culture containing 10 mM of unlabeled ammonium (Supplementary Table 3). According to the data above, less than 0.01 mM of N from 10 mM ammonium was converted to N_2O. Assuming 1 mM 10% ^{15}N labeled N-containing additives were entirely converted to 10% ^{15}N labeled N_2O, the amount of N_2O produced would be 100 times greater than that produced by ammonium. This explains why the final ^{15}N abundance of accumulated N_2O remains at 10%, unaffected by the unlabeled N_2O generated from unlabeled ammonium. To confirm this conjecture, we reduced the concentration of the 10% ^{15}N-labeled N-containing additive from 1mM to 0.05 mM. The immediate decrease in $^{15}N_2O$ abundance by nearly half (from 10% to around 5%) indicated that the amount of N_2O produced from 10 mM unlabeled ammonium was similar to that produced by 0.05 mM 10% ^{15}N labeled N-containing additive, which at most could account for 0.5% of the N content of 10 mM ammonium. Allowing for some margin of error in the data, we concluded that the amount of N_2O produced from ammonium did not exceed 1% of the initial ammonium. In summary, the results from three experiments (nitrite accumulation in the DDC experiment, N_2O accumulation, and ^{15}N abundance change in the acetylene experiment) collectively suggest that less than 1% of the ammonium underwent conversion through the nitrification and denitrification processes. As 40-50% ammonium could be converted to N_2 by *Z. taiwanensis* [5], these inhibiting experiments indicated that previously known nitrification and denitrification processes were not the primary pathways for the conversion of ammonium to N_2.

An important question arising from the N-containing additives experiments is why the majority of added inorganic N-containing additives could be converted to N_2O (Supplementary Table 3),

while only a small amount of ammonium was converted to N_2O if these additives were considered intermediates. The conversion amount of intermediates to N_2O should be quantitatively equivalent to the amount of N_2O generated from ammonium. This question was resolved after the discovery of acetoxime. The amount of N_2O produced upon the addition of acetoxime was found to be very small (0.43 μmol), consistent with the N_2O content (0.31 μmol) resulting from ammonium conversion, but distinct from tests involving other nitrogen compounds which generated excessive N_2O (Supplementary Table 3).

Trace nitrite production during aerobic ammonium oxidation process by *Z. taiwanensis*

Amo, *hao*, and *pod* like genes were not present in the genome of *Z. taiwanensis*, suggesting that trace nitrite production might not be an enzymatic process. In DDC experiments (Supplementary Fig. 1C), a small increase in nitrite concentration was observed with the presence of DDC when acetoxime was added under oxic conditions, and the amount of increased nitrite (<1% acetoxime-N) was consistent with the accumulated nitrite (<1% NH_4^+-N) from the ammonium. The nitrite produced by the control group without *Z. taiwanensis* was almost identical, suggesting that nitrite was produced by the abiotic oxidation of acetoxime under oxic conditions. This was further demonstrated by anoxic experiments showing that nitrite was not observed with the presence of DDC and Fe(III) under anoxic conditions, even when additional acetoxime was added. The slow nitrite production from acetoxime was also reported in earlier work [6] where 3-5% of acetoxime-N was nitrified by *Nocardia corallina* in peptone medium after 2 weeks, albert whether the nitrification process is biotic or not. The contribution of abiotic oxidation of nitrogen compounds was recently emphasized in *Alcaligenes* species [7], but our data suggest that the abiotic process which leads to minimal nitrite production do not play a significant role during ammonium oxidation by *Z. taiwanensis*.

Involvement of hydroxylamine during aerobic ammonium oxidation process by *Z. taiwanensis*

Acetoxime is known to be spontaneously synthesized from hydroxylamine and acetone under alkaline conditions through chemical reactions [8, 9]. Initially, it was hypothesized that *Z. taiwanensis* could convert ammonium to hydroxylamine, making it straightforward to generate acetoxime in the presence of acetone. However, when hydroxylamine and acetone were used as substrates, less amounts of acetoxime was produced in the presence of *Z. taiwanensis* compared to those without *Z. taiwanensis* (Fig. 2A). Furthermore, when 1 mM hydroxylamine was added to the bacteria culture with the presence of acetylene, significant production of N_2O (8.44 μmol) was observed which is different from the minimal N_2O production (0.43 μmol) after the addition of 1 mM acetoxime (Supplementary Table 3). No obvious abiotic production of $^{15}N_2O$ was detected when $^{15}NH_2OH$ was added in the control group without *Z. taiwanensis*, probably due to the alkaline conditions (pH > 8.5) [10]. Moreover, *Z. taiwanensis* was found to be incapable of utilizing or removing hydroxylamine (Supplementary Fig. 1D). The nitrite produced as the product of hydroxylamine oxidation was likely an abiotic process [6], as there was no distinguishable difference in the consumed hydroxylamine amounts and accumulated nitrite levels with or without *Z. taiwanensis*. These observations indicate that hydroxylamine is not involved in the biosynthesis of acetoxime.

Hydroxylamine can be generated through acetoxime hydrolysis under non-alkaline conditions as follows: $(CH_3)_2C{=}N{-}OH + H_2O \leftrightharpoons (CH_3)_2C{=}O + NH_2OH$. However, in our study on the ammonium oxidation process by *Z. taiwanensis*, hydroxylamine was not detected. One key factor that may have influenced the production of hydroxylamine is the pH value, which ranged from 8.5 to 10.0 during oxic incubation, a range distinct from the typically observed pH of around 7.0 in previous studies where hydroxylamine production from ammonium was reported in *Arthrobacter*

globiformis [11], *Alcaligenes faecalis* [7], and *Pseudomonas putida* [3].

Pathway for acetone production by *Z. taiwanensis*

Z. taiwanensis is capable of converting various organic compounds into acetone, with pyruvate demonstrating slightly higher conversion efficiency (Fig. 2E), possibly following the common pathway (pyruvate → acetyl-CoA → acetoacetyl-CoA → acetoacetate → acetone) [12]. However, butanol, which usually coexists with acetone in acetone-butanol-ethanol (ABE) fermentation, was not detected in this study. The biosynthesis of acetone from organic matter is a well-documented phenomenon, with numerous reports in both natural and engineered ecosystems [13-15]. The pathway for acetone production is diverse, involving decarboxylation of acetoacetate by acetoacetate decarboxylases (AAD) [16], the conversion of 2-propanol by alcohol dehydrogenases (ADH) [17], isomerization of epoxypropane [18], decomposition of 3-hydroxy-3-methylglutaryl-coenzyme A [19], and oxidation of 2-nitropropane or atrazine [20, 21]. To investigate the direct precursors for acetone production, acetoacetate, 2-propanol, and hydroxyacetone were added to cultures containing pyruvate as the carbon source. Significant acetone production was observed in both the 2-propanol and acetoacetate groups (Fig. 2F). The existence of three distinct secondary ADH in the genome of *Z. taiwanensis* may facilitate the conversion of 2-propanol. Although *Z. taiwanensis* can encode CoA transferase to catalyze acetoacetate production in the pyruvate pathway, its genome lacks known genes encoding AAD. The presence of two unknown proteins belonging to the fumarylacetoacetate hydrolase family protein (AAD also belongs to this family) might catalyze this reaction. Furthermore, *Z. taiwanensis* possesses other proteins outside the common pathway that can catalyze acetoacetate production, such as fumarylacetoacetase and hydroxymethylglutaryl-CoA lyase. The acetone production pathway in *Z. taiwanensis* is not singular and likely depends on the substrate utilized.

Homologs of AOS and AOH

NCBI BLASTP searches detected AOS and AOH proteins respectively in thousands of prokaryotic and eukaryotic genomes from *Acidobacteria* (298 and 1197 hits), *Actinobacteria* (1624 and 1825 hits), *Chloroflexi* (357 and 1287 hits), *Chrysiogenota* (11 and 37 hits), *Cyanobacteria* (750 and 959 hits), *Deinococcota* (16 and 1849 hits), FCB group bacteria (117 and 1575 hits), *Firmicutes* (158 and 1980 hits), *Gemmatimonadetes* (134 and 1375 hits) , *Lentisphaerae* (23 and 976 hits), *Nitrospirae* (181 and 1577 hits), *Planctomycetes* (130 and 1290 hits), *Proteobacteria* (5379 and 6757 hits), *Spirochaetae* (860 and 1166 hits), *Thermodesulfobacteria* (460 and 1332 hits), *Verrucomicrobia* (703 and 1316 hits), *Archaea* (109 and 1577 hits), *Fungi* (1418 and 1359 hits), *Isopoda* (14 and 183 hits) and several eukaryotic algae. Various archaea and eukaryotic organisms especially fungi were found to possess AOS and AOH homologs, revealing that AMAO were not just involved in the bacteria.

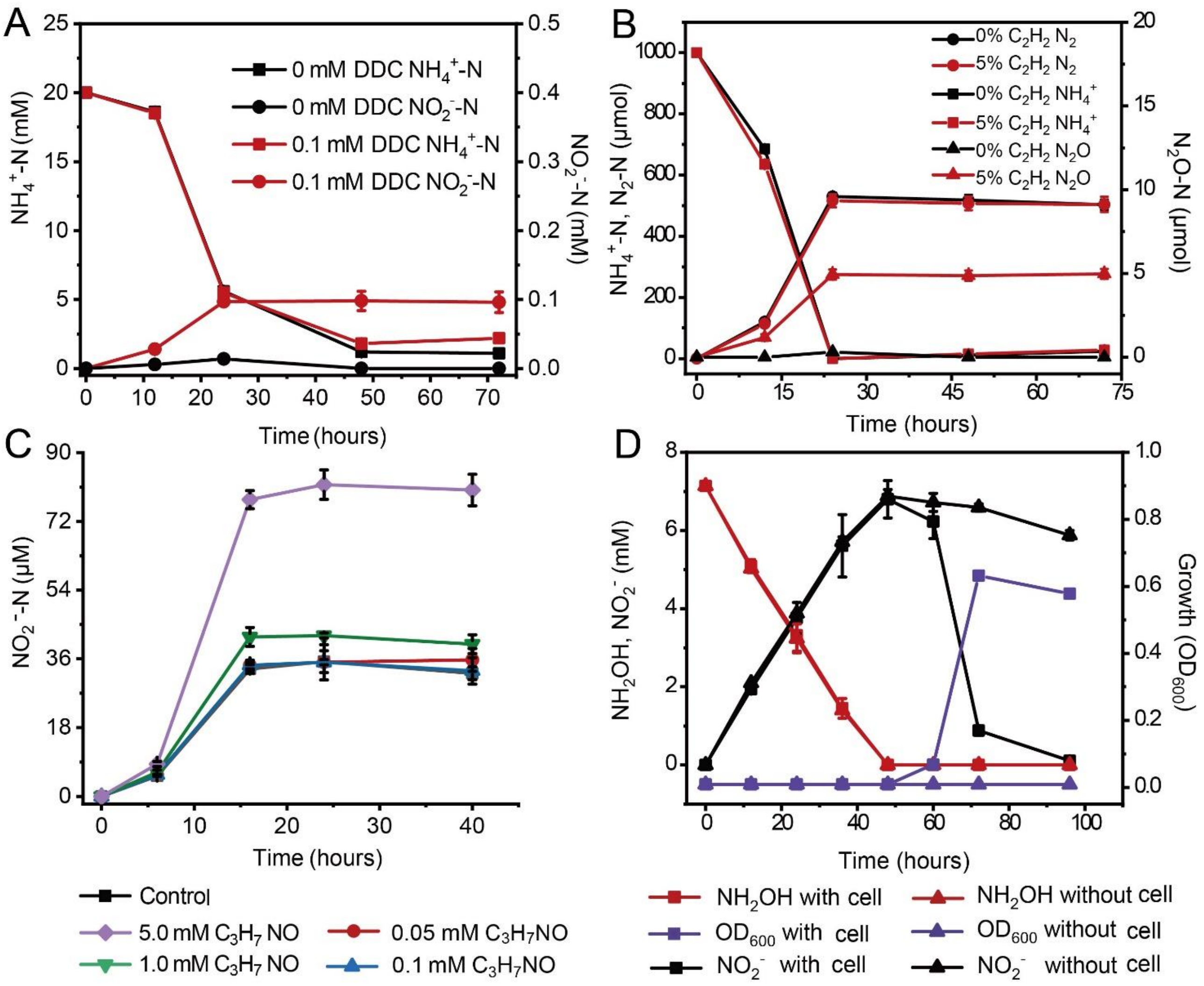

Supplementary Fig. 1

Involvement of nitrous oxide, nitrite, and hydroxylamine during ammonium removal by *Z. taiwanensis*. (A) Influence of DDC on NO_2^- production and NH_4^+ removal by *Z. taiwanensis* under oxic conditions. (B) Influence of acetylene (C_2H_2) on N_2O and N_2 production during ammonium removal by *Z. taiwanensis* under oxic conditions. (C) NO_2^- production with the presence of DDC and various concentrations of acetoxime (C_3H_7NO) in the *Z. taiwanensis* culture under oxic conditions. Control denotes no acetoxime added. (D) Utilization of NH_2OH by *Z. taiwanensis*. Data points represent means, error bars show 1 s.d. of n = 3 biological replicates.

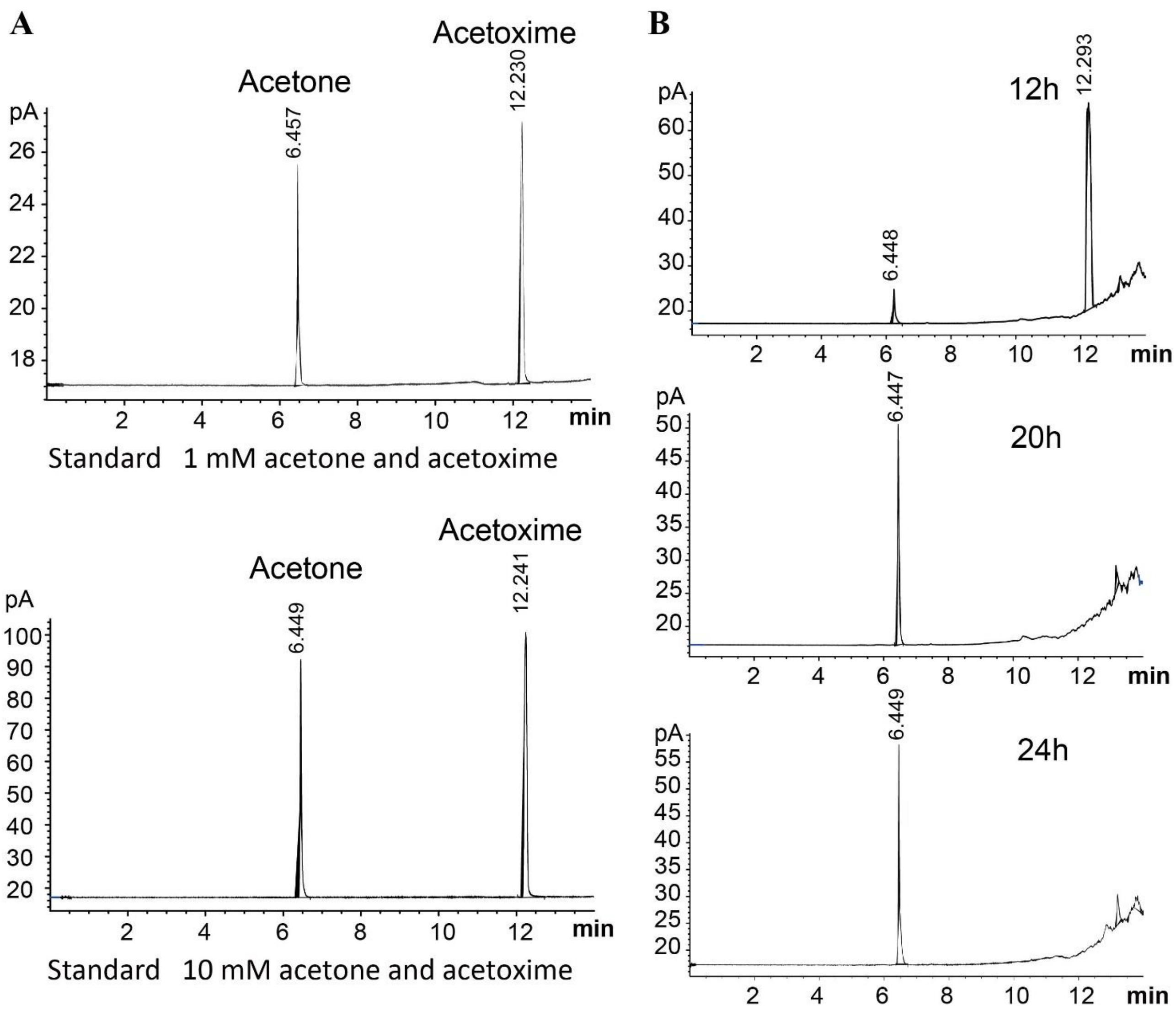

Supplementary Fig. 2

GC analysis of acetone and acetoxime production by *Z. taiwanensis*. (A) Chromatograms of 1 mM and 10 mM acetone and acetoxime in fresh BM, showing retention times (indicated by peak labels) for reference. (B) Detection of acetone and acetoxime in BM from oxic incubations with acetylene. Peaks correspond to retention times observed in (A), confirming metabolite identity. Values above peaks denote retention times (in minutes).

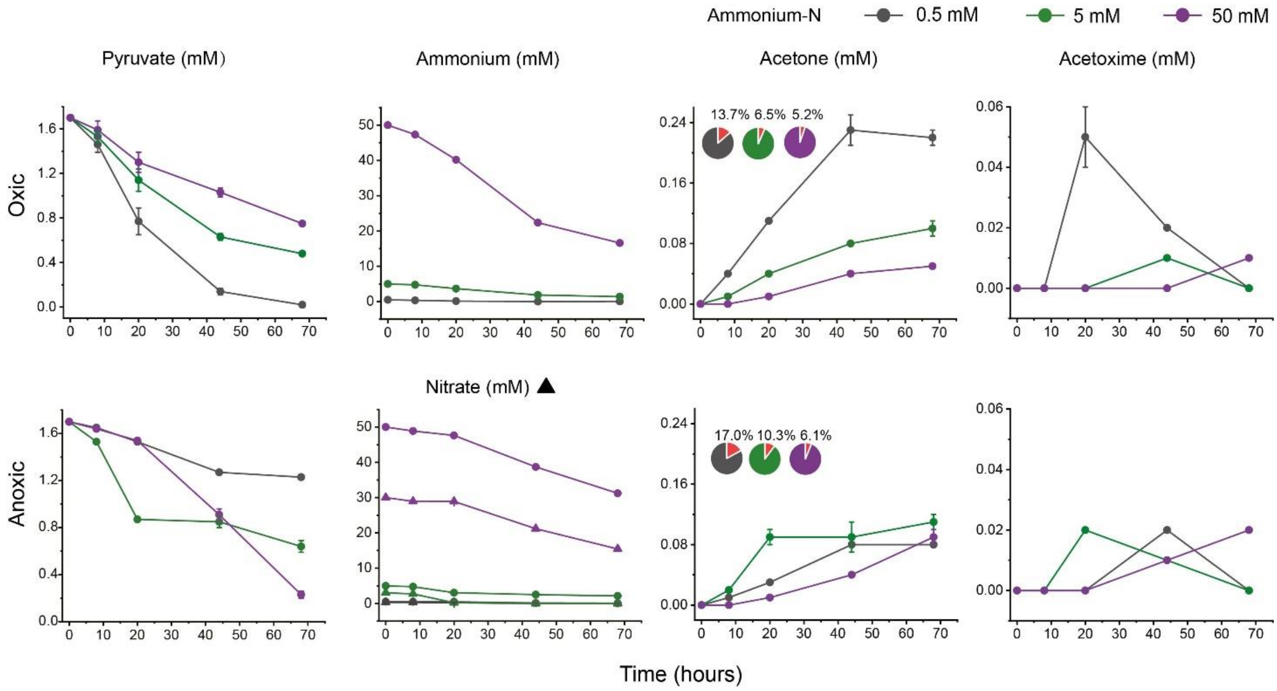

Supplementary Fig. 3

Effects of 5 mM Pyruvate-C and varying ammonium-N concentrations on the AMAO process by *Z. taiwanensis* under oxic and anoxic conditions. 5 mM Pyruvate-C and ammonium-N (0.5, 5, and 50 mM) were tested. Acetone production percentages relative to pyruvate consumption are shown as color-coded pie charts. Data are means ±1 s.d. (n = 3 biological replicates).

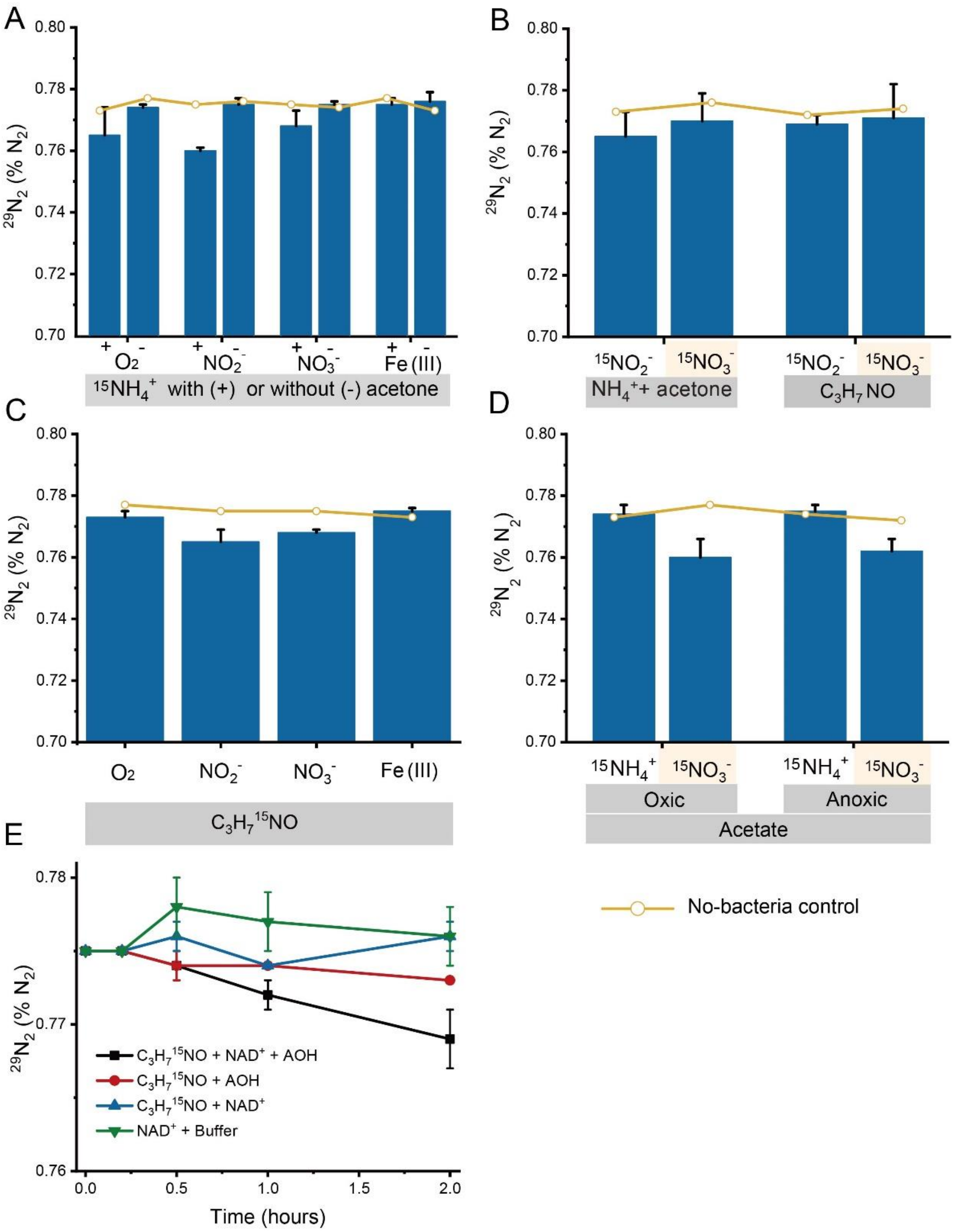

Supplementary Fig. 4

$^{29}N_2$ measurements in $^{15}N_2$ detection experiments with *Z.taiwanensis* cells or purified AOH enzymes. $^{29}N_2$ (% N_2) means the mole fraction of $^{29}N_2$ in total N_2. (A) ^{15}N-labeled ammonium with or without acetone was treated with O_2 under oxic conditions (He/O_2=1:1) and NO_2^-, NO_3^-, or Fe(III) under anoxic conditions (pure He). (B) Non-^{15}N-labeled ammonium or acetoxime (C_3H_7NO) were treated with ^{15}N-labeled NO_2^- or NO_3^-. (C) ^{15}N-labeled acetoxime was treated with O_2 under oxic conditions and NO_2^-, NO_3^-, or Fe(III) under anoxic conditions. (D) ^{15}N-labeled ammonium or nitrate was treated with acetate under oxic or anoxic conditions. (E) Production of $^{29}N_2$ in purified AOH assays.

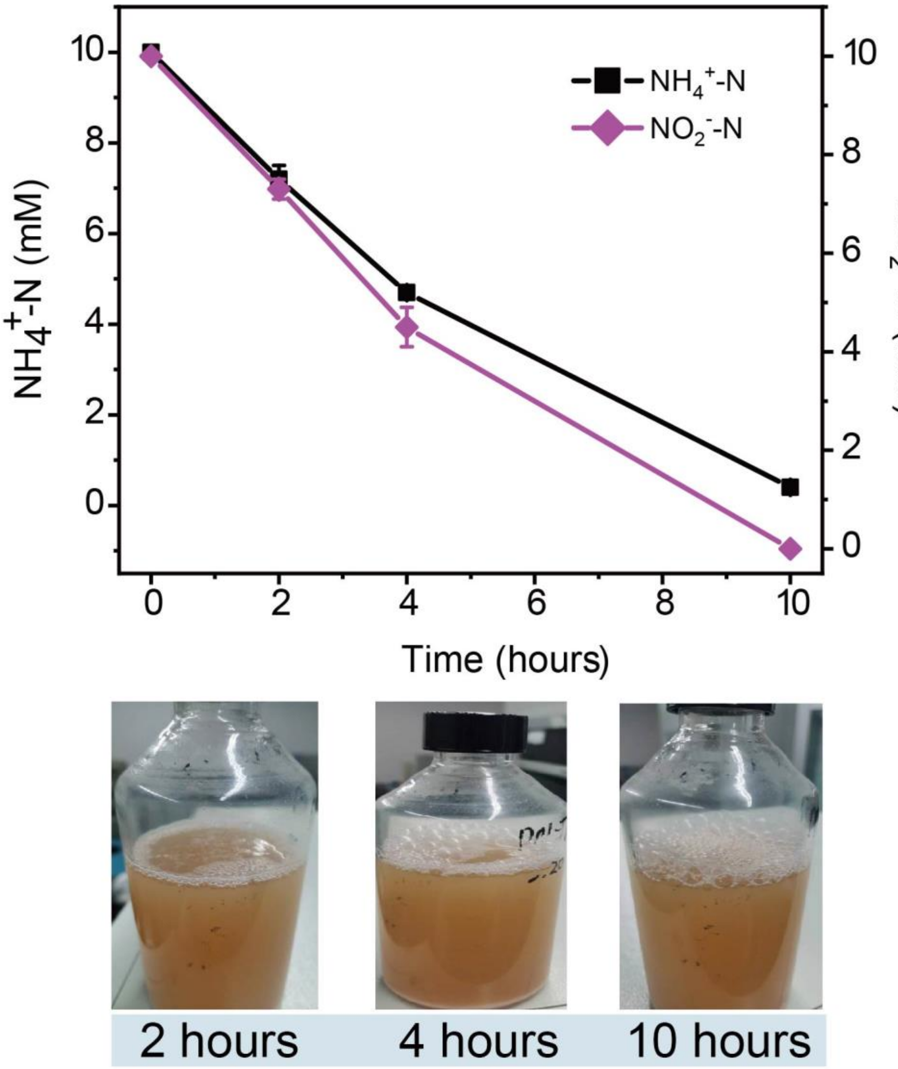

Supplementary Fig. 5

Simultaneous removal of 10 mM equivalent of ammonium and nitrite in the presence of 10 mM acetone by *Z. taiwanensis*. Harvested fresh cells were suspended with a modified medium devoid of any carbon and nitrogen sources (final OD_{600}=2.24). Acetone, ammonium and nitrite were subsequently added, and the bottles were incubated without agitation. The photos at the bottom of the figure were captured at different time points corresponding to the sampling times. Data points represent means, error bars show 1 s.d. of n = 3 biological replicates.

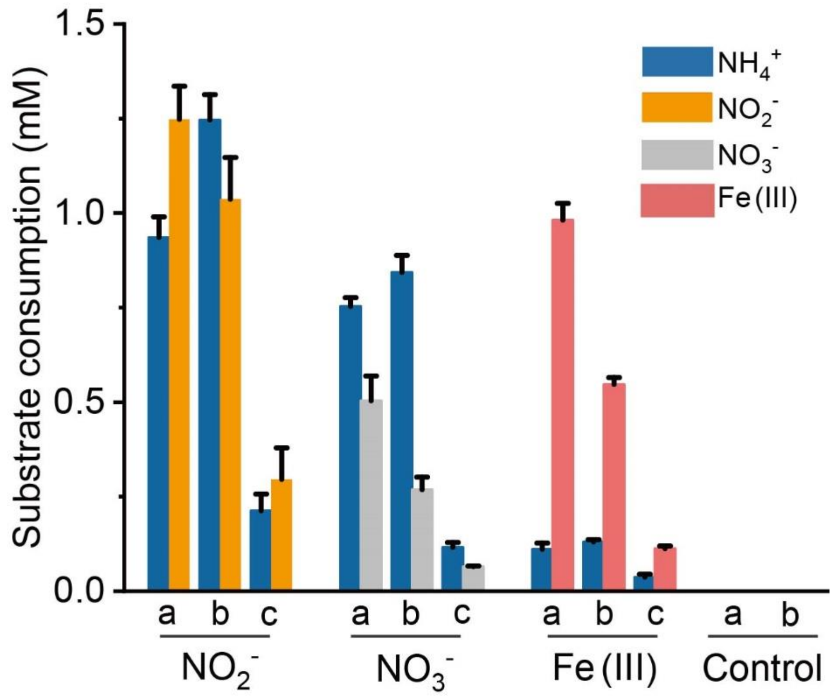

Supplementary Fig. 6

Impact of NAD^+/NADH redox cycle on the connection between ammonium oxidation and nitrate, nitrite, or ferric reduction. 0.1 g of *Z. taiwanensis* cell-free extract was added to 2 ml reaction mixtures containing 50 mM Tris-HCl (pH 8.5), 5 mM NH_4Cl, 5 mM acetone, 3 mM NAD^+ (a) or NADH (b) or nothing (c), and 5 mM $NaNO_2$, $NaNO_3$, or ferric citrate. Control experiments without cell-free extract were also conducted; n=3 (error bars, s.d.).

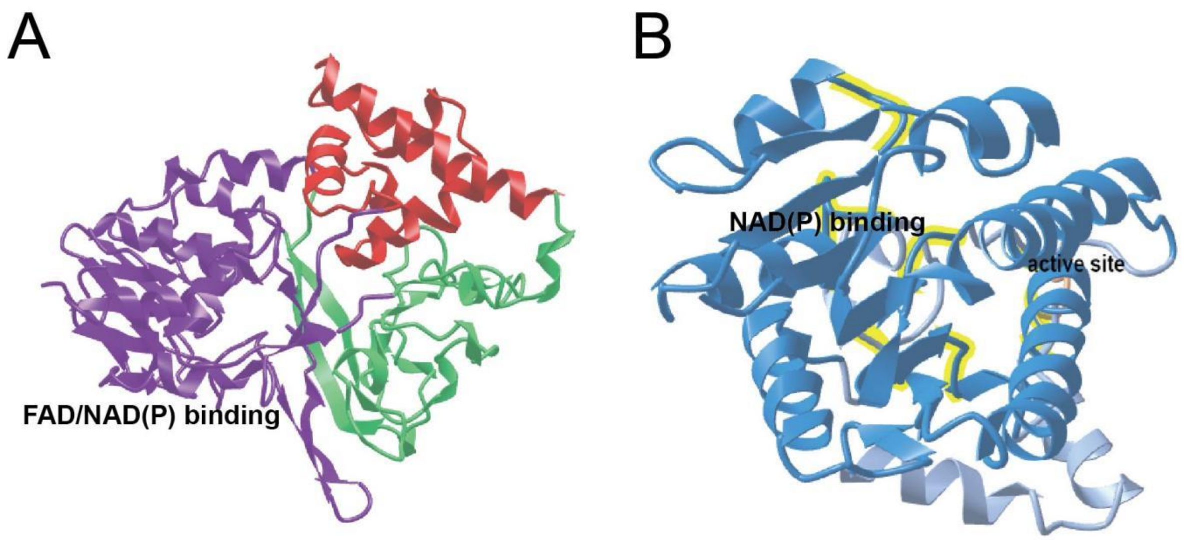

Supplementary Fig. 7

Predicted structures of AOS (A) and AOH (B). All images were prepared via two protein databases, the AlphaFold Protein Structure Database and the InterPro Protein Database.

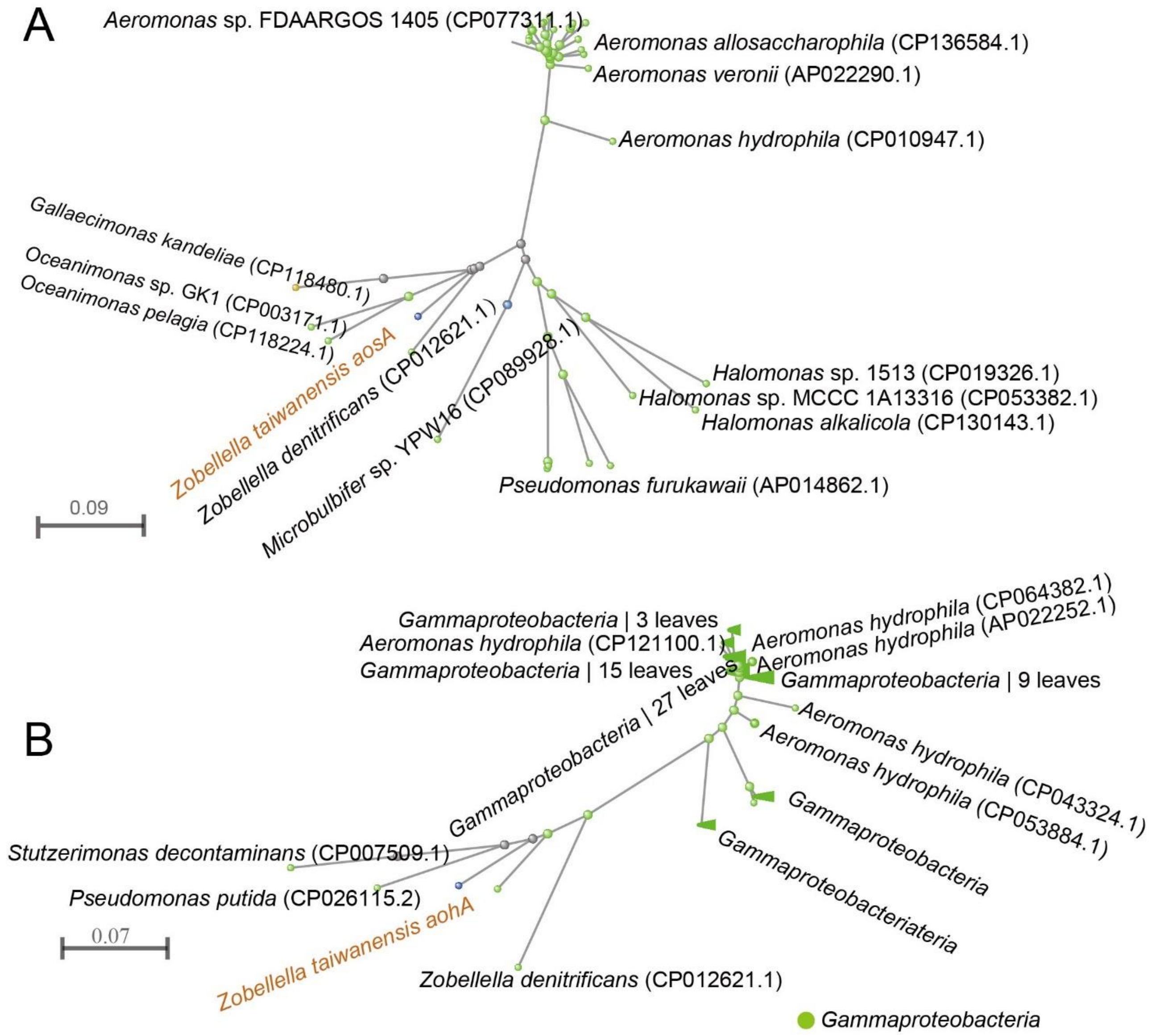

Supplementary Fig. 8

Phylogenetic affiliation of *aosA* and *aohA* sequences to other homologous gene sequences from various strains. A Neighbor-joining trees showing *aosA* (A) and *aohA* (B) phylogenies. The scale bars represent 0.09 (A) and 0.07 (B) estimated substitutions per residue.

Supplementary Table 1.

Substrate combinations and their associated reactions in the study

Substrate combination	Targeted reaction/Equations No.
$^{15}NH_4^+ + C_3H_6O + O_2$	$NH_4^+ + (CH_3)_2C{=}O \rightarrow (CH_3)_2C{=}N\text{-}OH + 2e^- + 3H^+$ (1)<br>$(CH_3)_2C{=}N\text{-}OH \rightarrow 0.5N_2 + (CH_3)_2C{=}O + e^- + H^+$ (2)<br>$3H^+ + 3e^- + 0.75O_2 \rightarrow 1.5H_2O$ (3)<br>$NH_4^+ + 0.75O_2 \rightarrow 0.5N_2 + 1.5H_2O + H^+$ (4)
$^{15}NH_4^+ + C_3H_6O + {}^{14}NO_2^-$<br>$^{14}NH_4^+ + C_3H_6O + {}^{15}NO_2^-$	$NH_4^+ + (CH_3)_2C{=}O \rightarrow (CH_3)_2C{=}N\text{-}OH + 2e^- + 3H^+$ (1)<br>$(CH_3)_2C{=}N\text{-}OH \rightarrow 0.5N_2 + (CH_3)_2C{=}O + e^- + H^+$ (2)<br>$4H^+ + 3e^- + NO_2^- \rightarrow 2H_2O + 0.5N_2$ (5)<br>$NH_4^+ + NO_2^- \rightarrow N_2 + 2H_2O$ (6)
$^{15}NH_4^+ + C_3H_6O + {}^{14}NO_3^-$<br>$^{14}NH_4^+ + C_3H_6O + {}^{15}NO_3^-$	$NH_4^+ + (CH_3)_2C{=}O \rightarrow (CH_3)_2C{=}N\text{-}OH + 2e^- + 3H^+$ (1)<br>$(CH_3)_2C{=}N\text{-}OH \rightarrow 0.5N_2 + (CH_3)_2C{=}O + e^- + H^+$ (5)<br>$6H^+ + 5e^- + NO_3^- \rightarrow 3H_2O + 0.5N_2$ (7)<br>$NH_4^+ + 0.6NO_3^- \rightarrow 0.8N_2 + 0.4H^+ + 1.8H_2O$ (8)
$^{15}NH_4^+ + C_3H_6O + Fe(III)$	$NH_4^+ + (CH_3)_2C{=}O \rightarrow (CH_3)_2C{=}N\text{-}OH + 2e^- + 3H^+$ (1)<br>$(CH_3)_2C{=}N\text{-}OH \rightarrow 0.5N_2 + (CH_3)_2C{=}O + e^- + H^+$ (2)<br>$e^- + Fe^{3+} \rightarrow Fe^{2+}$ (9)<br>$NH_4^+ + 3Fe^{3+} \rightarrow 0.5N_2 + 4H^+ + 3Fe^{2+}$ (10)
$C_3H_7{}^{15}NO + O_2$	$(CH_3)_2C{=}N\text{-}OH + 0.25O_2 \rightarrow 0.5N_2 + (CH_3)_2C{=}O + 0.5H_2O$<br>(2)(3)
$C_3H_7{}^{15}NO + {}^{14}NO_2^-$	$3(CH_3)_2C{=}N\text{-}OH + NO_2^- \rightarrow 2N_2 + 3(CH_3)_2C{=}O + H_2O + OH^-$<br>(2)(5)
$C_3H_7{}^{14}NO + {}^{15}NO_2^-$	Same as above, tracking ^{15}N in NO_2^-
$C_3H_7{}^{15}NO + {}^{14}NO_3^-$	$5(CH_3)_2C{=}N\text{-}OH + NO_3^- \rightarrow 3N_2 + 5(CH_3)_2C{=}O + 2H_2O + OH^-$<br>(2)(7)
$C_3H_7{}^{14}NO + {}^{15}NO_3^-$	Same as above, tracking ^{15}N in NO_3^-
$C_3H_7{}^{15}NO + Fe(III)$	$(CH_3)_2C{=}N\text{-}OH + Fe^{3+} \rightarrow 0.5N_2 + (CH_3)_2C{=}O + Fe^{2+} + H^+$<br>(2)(10)
$^{15}NH_4^+ + C_3H_6O$	$NH_4^+ + (CH_3)_2C{=}O \rightarrow (CH_3)_2C{=}N\text{-}OH + 2e^- + 3H^+$ (1)<br>without e^- acceptor
$C_3H_7{}^{15}NO$	$(CH_3)_2C{=}N\text{-}OH \rightarrow 0.5N_2 + (CH_3)_2C{=}O + e^- + H^+$ (2)<br>without e^- acceptor
$^{15}NH_4^+ + O_2$<br>$^{15}NH_4^+ + {}^{14}NO_2^-$<br>$^{15}NH_4^+ + {}^{14}NO_3^-$<br>$^{15}NH_4^+ + Fe(III)$	$NH_4^+ + 0.75O_2 \rightarrow 0.5N_2 + 1.5H_2O + H^+$ without acetone (4)<br>$NH_4^+ + NO_2^- \rightarrow N_2 + 2H_2O$ without acetone (6)<br>$NH_4^+ + 0.6NO_3^- \rightarrow 0.8N_2 + 0.4H^+ + 1.8H_2O$ without acetone (8)<br>$NH_4^+ + 3Fe^{3+} \rightarrow 0.5N_2 + 4H^+ + 3Fe^{2+}$ without acetone (10)
$^{15}NH_4^+ + CH_3COONa + O_2$<br>$^{15}NH_4^+ + CH_3COONa$	Ammonium oxidation with acetate (4)<br>Ammonium oxidation with acetate (no O_2) (4)
$^{15}NO_3^- + CH_3COONa + O_2$	$6H^+ + 5e^- + NO_3^- \rightarrow 3H_2O + 0.5N_2$ (aerobic denitrification) (7)
$^{15}NO_3^- + CH_3COONa$	$6H^+ + 5e^- + NO_3^- \rightarrow 3H_2O + 0.5N_2$ (anaerobic denitrification)<br>(7)

Supplementary Table 2

Primers used in this study

Name	Sequence（5’ to 3’）	Description
pBAD-F	AAGCTTGGCTGTTTTGGCGG	For vector linaering
PBAD-R	GGTTAATTCCTCCTGTTAGCCCAAAAA	
aohA-F	GGGCTAACAGGAGGAATTAACCATGAAGCGCGTACTCATTACCG	For PCR *aohA* gene
aohA-R	CCGCCAAAACAGCCAAGCTTTTACTCATGCTTGCTCCTTGTCATG	
aosA-F	GGGCTAACAGGAGGAATTAACCATGAGTAACATCGCCGTGATCG	For PCR *aosA* gene
aosA-R	CCGCCAAAACAGCCAAGCTTTCATAGCGCCTCCCCGAAG	
hypA-F	GGGCTAACAGGAGGAATTAACCATGAGCCTTAACAGTGGCA	For PCR *hypA* gene
hypA-R	CCGCCAAAACAGCCAAGCTTTCAGGGATGTGGATGATAGG	

Supplementary Table 3

Determination of ^{15}N abundance and quantity of N_2O produced by *Z. taiwanensis* under oxic conditions in multiple experiments. Acetylene was used as an inhibitor to halt N_2O reduction in all experiments except the control without C_2H_2. The initial ^{15}N abundance ($^{15}N\%$) of all traced nitrogen compounds is about 10%. + $C_3H_7{}^{15}NO$ (^{15}N labeled acetoxime) indicates that $C_3H_7{}^{15}NO$ was added as a testing intermediate for ammonium oxidation, while the other groups were similar. Testing intermediates containing 1 mM nitrogen were added in group A and 0.05 mM nitrogen were used in group B. ND means not detected. Data are averages from three independent experiments.

Experiment design	A group		B group	
	N_2O ($^{15}N\%$)	N_2O (μmol)	N_2O ($^{15}N\%$)	N_2O (μmol)
$^{15}NH_4$ without C_2H_2	0.37	ND		
$^{15}NH_4$	10.00±0.44	0.31±0.02		
$^{14}NH_4^+$+$C_3H_7{}^{15}NO$	1.84±0.33	0.43±0.03	0.37	0.35±0.01
$^{14}NH_4^+$+$^{15}NO_2^-$	10.29±0.18	9.65±0.05	5.73±0.08	0.72±0.03
$^{14}NH_4^+$+$^{15}NH_2OH$	11.34±0.05	8.44±0.16	5.14±0.11	0.67±0.01
$^{14}NH_4^+$+^{15}NO	10.79±0.02	9.33±0.09	5.90±0.05	0.78±0.02
$^{14}NH_4^+$+$^{15}N_2O$	11.69±0.09	10.06±0.13	6.00±0.07	0.85±0.14
$^{15}NO_3^-$	10.09±0.06	63.24±0.32		

Supplementary Table 4
General features of examined genome

	Z. taiwanensis
Sequence size (bp)	3,812,818
GC content (%)	61.9
Number of contigs	1
N50 (bp)	24,057,000
Number of coding sequences	3,462
Number of rRNA	25
Number of tRNA	97
Proteobacteria Lineage	γ
Number of subsystems	499

Supplementary Table 5
Inserted complete gene fragments in the AC3 clone

Start	Stop	Function
3103330	3102305	Transcriptional regulator, LacI family
3103562	3104569	Myo-inositol 2-dehydrogenase 1 (EC 1.1.1.18)
3104699	3105622	Inositol transport system sugar-binding protein
3106711	3105701	Transmembrane transporter, major facilitator family
3106919	3106728	Transmembrane transporter, major facilitator family
3107088	3107933	Transcriptional regulator, AraC family
3108103	3108924	5-deoxy-glucuronate isomerase (EC 5.3.1.-)
3108967	3109953	Myo-inositol 2-dehydrogenase 1 (EC 1.1.1.18)
3110192	3111007	Hydroxypyruvate isomerase (EC 5.3.1.22)
3111195	3112148	COG1683: Uncharacterized conserved protein / FIG143828: Hypothetical protein YbgA
3112138	3112983	Transcriptional regulator, MerR family, associated with photolyase
3112955	3114364	Deoxyribodipyrimidine photolyase (EC 4.1.99.3)
3114361	3114783	FIG002994: Putative transcriptional regulator
3114780	3115511	NAD (P)-dependent oxidoreductase, short-chain dehydrogenase/reductase family (EC 1.1.1.-)
3115504	3116751	FAD-dependent oxidoreductase, COG2907: Amine oxidase, flavin-containing
3116748	3117467	FIG001571: Hypothetical protein
3117480	3118733	S-adenosyl-L-methionine dependent methyltransferase, similar to cyclopropane-fatty-acyl-phospholipid synthase
3118790	3119191	FIG024285: Hypothetical protein
3119188	3119670	FIG026291: Hypothetical periplasmic protein
3119672	3120193	FIG002577: Putative lipoprotein precursor
3120783	3120190	Nucleotidase YfbR, HD superfamily
3120852	3121148	protein of unknown function DUF1244
3122121	3121231	Formyltetrahydrofolate deformylase (EC 3.5.1.10)
3122566	3122108	UPF0225 protein YchJ
3123631	3122675	18K peptidoglycan-associated outer membrane lipoprotein; Peptidoglycan-associated lipoprotein precursor; Outer membrane protein P6; OmpA/MotB precursor
3124029	3124289	C protein
3124310	3125182	Zn-dependent protease with chaperone function PA4632
3125191	3126111	Latent glucokinase ycfX
3126163	3126774	FKBP-type peptidyl-prolyl cis-trans isomerase FklB (EC 5.2.1.8)
3126914	3127726	4-hydroxy-tetrahydrodipicolinate reductase (EC 1.17.1.8)
3128246	3129277	Carbamoyl-phosphate synthase small chain (EC 6.3.5.5)
3129297	3132524	Carbamoyl-phosphate synthase large chain (EC 6.3.5.5)
3132797	3133000	Cold shock protein CspD
3133553	3133137	Putative Holliday junction resolvase YqgF
3134104	3133550	UPF0301 protein YqgE
3135123	3134176	Glutathione synthetase (EC 6.3.2.3)

3135752	3135120	Ribosomal RNA small subunit methyltransferase E (EC 2.1.1.-)
3136533	3135829	Endonuclease I
3137107	3136637	Antioxidant, putative
3137615	3137121	Protein sprT
3138823	3137666	S-adenosylmethionine synthetase (EC 2.5.1.6)
3139066	3141063	Transketolase (EC 2.2.1.1)
3144094	3144240	Hypothetical protein
3144117	3141160	Diguanylate cyclase/phosphodiesterase (GGDEF & EAL domains) with PAS/PAC sensor(s)
3144289	3144708	Probable transmembrane protein

Supplementary Table 6

Annotation table of the genes indicated in the circular genome map

Location	Strand	Function	Abbreviation
1|quiver_525438_526859	+	Copper-containing nitrite reductase	NirK
1|quiver_915474_916190	+	Ferric siderophore transport system, periplasmic binding protein	TonB
1|quiver_916194_916817	+	Ferric siderophore transport system, biopolymer transport protein	ExbB
1|quiver_1115236_1114913	-	Iron dicitrate transport protein	FecA
1|quiver_1186309_1185296	-	Ferric iron ABC transporter, iron-binding protein	
1|quiver_1187760_1186534	-	Ammonium transporter	
1|quiver_1188114_1187776	-	Nitrogen regulatory protein	
1|quiver_1509586_1508750	-	ABC-type Fe^{3+}-siderophore transport system, ATPase component	ABC-type Fe^{3+}-siderophore
1|quiver_1510659_1509583	-	ABC-type Fe^{3+}-siderophore transport system, permease 2 component	ABC-type Fe^{3+}-siderophore
1|quiver_1511660_1510656	-	ABC-type Fe^{3+}-siderophore transport system, permease component	ABC-type Fe^{3+}-siderophore
1|quiver_1512601_1511660	-	ABC-type Fe^{3+}-siderophore transport system, periplasmic iron-binding component	ABC-type Fe^{3+}-siderophore
1|quiver_1528173_1530449	+	Nitric-oxide reductase, quinol-dependent	NorB
1|quiver_1602030_1600141	-	Nitrous-oxide reductase	NosZ
1|quiver_1916126_1914699	-	Nitrogen regulation protein	NR(I)
1|quiver_1918984_1917419	-	Glutamine synthetase type I	GlnA
1|quiver_2371610_2371275	-	Nitrite reductase [NAD(P)H] small subunit	
1|quiver_2374120_2371607	-	Nitrite reductase [NAD(P)H] large subunit	
1|quiver_2376078_2375266	-	Nitrate ABC transporter, ATP-binding protein	
1|quiver_2376982_2376089	-	Nitrate ABC transporter, permease protein	
1|quiver_2378441_2377074	-	Nitrate ABC transporter, nitrate-binding protein	
1|quiver_2499755_2500093	+	Nitrogen regulatory protein	P-II
1|quiver_2701850_2701065	-	Iron dicitrate transport ATP-binding protein	FecE
1|quiver_2702905_2701844	-	Iron dicitrate transport system permease protein	FecD
1|quiver_2849488_2847527	-	Ferric hydroxamate ABC transporter, permease component	FhuB

1|quiver_2851164_2850397	-	Ferrichrome transport ATP-binding protein	FhuC
1|quiver_3114780_3115511	+	NAD(p)-dependent oxidoreductase, short-chain dehydrogenase	AohA
1|quiver_3115504_3116751	+	FAD-dependent oxidoreductase, COG2907: Amine oxidase, flavin-containing	AosA
1|quiver_3212013_3212492	+	Urease accessory protein	UreE
1|quiver_3391085_3390594	-	Ferric uptake regulation protein	Fur
1|quiver_3423730_3424230	+	Ferredoxin-type protein (periplasmic nitrate reductase)	NapF
1|quiver_3424227_3424499	+	Periplasmic nitrate reductase component	NapD
1|quiver_3424496_3426985	+	Nitrate reductase catalytic subunit	NapA
1|quiver_3426993_3427739	+	Ferredoxin-type protein (periplasmic nitrate reductase)	NapG
1|quiver_3427741_3428607	+	Polyferredoxin (periplasmic nitrate reductase)	NapH
1|quiver_3429078_3429671	+	Cytochrome c-type protein	NapC

Supplementary References

1. W. M. Haynes. *Handbook of Chemistry and Physics.* CRC Press, ed.97th, 2016-2017.
2. Moir JWB, Baratta D, Richardson DJ *et al.* The purification of a cd1-type nitrite reductase from, and the absence of a copper-type nitrite reductase from, the aerobic denitrifier thiosphaera-pantotropha - the role of pseudoazurin as an electron-donor. *Eur J Biochem*. 1993;**212**:377-85 https://doi.org/10.1111/j.1432-1033.1993.tb17672.x
3. Daum M, Zimmer W, Papen H *et al.* Physiological and molecular biological characterization of ammonia oxidation of the heterotrophic nitrifier *Pseudomonas putida*. *Curr Microbiol*. 1998;**37**:281-88 https://doi.org/10.1007/s002849900379
4. McCarty GW. Modes of action of nitrification inhibitors. *Biol Fert Soils*. 1999;**29**:1-9 https://doi.org/10.1007/s003740050518
5. Lei Y, Wang YQ, Liu HJ *et al.* A novel heterotrophic nitrifying and aerobic denitrifying bacterium, *Zobellella taiwanensis* DN-7, can remove high-strength ammonium. *Appl Microbiol Biotechnol*. 2016;**100**:4219-29 https://doi.org/10.1007/s00253-016-7290-5
6. Jensen HL. Nitrification of oxime compounds by heterotrophic bacteria. *J Gen Microbiol*. 1951;**5**:360-68 https://doi.org/10.1099/00221287-5-2-360
7. Lenferink WB, Bakken LR, Jetten MSM *et al.* Hydroxylamine production by *Alcaligenes faecalis* challenges the paradigm of heterotrophic nitrification. *Sci Adv*. 2024;**10**:eadl3587 https://doi.org/10.1126/sciadv.adl3587
8. Liang XH, Mi ZT, Wang YQ *et al.* Synthesis of acetone oxime through acetone ammoximation over TS-1. *React Kinet Catal L*. 2004;**82**:333-37 https://doi.org/10.1023/B:REAC.0000034845.65961.3e
9. Wu JJ, Larsen SC. Solid-state nuclear magnetic resonance study of acetone oxime adsorbed on CuZSM-5 and on HZSM-5. *J Catal*. 1999;**182**:244-56 https://doi.org/10.1006/jcat.1998.2340
10. Su QX, Domingo-Félez C, Jensen MM *et al.* Abiotic nitrous oxide (N_2O) production is strongly pH dependent, but contributes little to overall N_2O emissions in biological nitrogen removal systems. *Environ Sci Technol*. 2019;**53**:3508-16 https://doi.org/10.1021/acs.est.8b06193
11. Gunner HB. Nitrification by *Arthrobacter globiformis*. *Nature*. 1963;**197**:1127-& https://doi.org/10.1038/1971127a0
12. Jones DT, Woods DR. Acetone-butanol fermentation revisited. *Microbiol Rev*. 1986;**50**:484-524 https://doi.org/10.1128/Mmbr.50.4.484-524.1986
13. Jacob DJ, Field BD, Jin EM *et al.* Atmospheric budget of acetone. *J Geophys Res-Atmos*. 2002;**107**:4100 https://doi.org/10.1029/2001JD000694
14. Wu BZ, Feng TZ, Sree U *et al.* Sampling and analysis of volatile organics emitted from wastewater treatment plant and drain system of an industrial science park. *Anal Chim Acta*. 2006;**576**:100-11 https://doi.org/10.1016/j.aca.2006.03.057
15. Schade GW, Custer TG. OVOC emissions from agricultural soil in northern Germany during the 2003 European heat wave. *Atmos Environ*. 2004;**38**:6105-14 https://doi.org/10.1016/j.atmosenv.2004.08.017
16. Tagaki W, Westheimer FH. Acetoacetate decarboxylase . Catalysis of hydrogen-deuterium exchange in acetone. *Biochemistry-Us*. 1968;**7**:901-+ https://doi.org/10.1021/bi00843a003
17. Kotani T, Yurimoto H, Kato N *et al.* Novel acetone metabolism in a propane-utilizing bacterium, *Gordonia* sp strain ty-5. *J Bacteriol*. 2007;**189**:886-93 https://doi.org/10.1128/Jb.01054-06
18. Small FJ, Tilley JK, Ensign SA. Characterization of a new pathway for epichlorohydrin degradation by whole cells of *Xanthobacter* strain Py2. *Appl Environ Microbiol*. 1995;**61**:1507-13 https://doi.org/10.1128/Aem.61.4.1507-1513.1995
19. Nemecek-Marshall M, Wojciechowski C, Wagner WP *et al.* Acetone formation in the vibrio family: A new pathway for bacterial leucine catabolism. *J Bacteriol*. 1999;**181**:7493-99

https://doi.org/10.1128/Jb.181.24.7493-7499.1999

20. Nagpal A, Valley MP, Fitzpatrick PF *et al.* Crystal structures of nitroalkane oxidase: Insights into the reaction mechanism from a covalent complex of the flavoenzyme trapped during turnover. *Biochemistry-Us*. 2006;**45**:1138-50 https://doi.org/10.1021/bi051966w

21. Nagy I, Compernolle F, Ghys K *et al.* A single cytochrome-P-450 system is involved in degradation of the herbicides eptc (s-ethyl dipropylthiocarbamate) and atrazine by *Rhodococcus* sp strain NI86/21. *Appl Environ Microbiol*. 1995;**61**:2056-60 https://doi.org/10.1128/AEM.61.5.2056-2060.1995
